# Natural variation in the plant polyadenylation complex

**DOI:** 10.3389/fpls.2023.1303398

**Published:** 2024-01-22

**Authors:** Lichun Zhou, Kai Li, Arthur G. Hunt

**Affiliations:** Department of Plant and Soil Sciences, University of Kentucky, Lexington, KY, United States

**Keywords:** alternative polyadenyaltion, natural variation, positive selection, *Arabidopsis* 1001 genomes, pseudogenes

## Abstract

Messenger RNA polyadenylation, the process wherein the primary RNA polymerase II transcript is cleaved and a poly(A) tract added, is a key step in the expression of genes in plants. Moreover, it is a point at which gene expression may be regulated by determining the functionality of the mature mRNA. Polyadenylation is mediated by a complex (the polyadenylation complex, or PAC) that consists of between 15 and 20 subunits. While the general functioning of these subunits may be inferred by extending paradigms established in well-developed eukaryotic models, much remains to be learned about the roles of individual subunits in the regulation of polyadenylation in plants. To gain further insight into this, we conducted a survey of variability in the plant PAC. For this, we drew upon a database of naturally-occurring variation in numerous geographic isolates of *Arabidopsis thaliana*. For a subset of genes encoding PAC subunits, the patterns of variability included the occurrence of premature stop codons in some *Arabidopsis* accessions. These and other observations lead us to conclude that some genes purported to encode PAC subunits in *Arabidopsis* are actually pseudogenes, and that others may encode proteins with dispensable functions in the plant. Many subunits of the PAC showed patterns of variability that were consistent with their roles as essential proteins in the cell. Several other PAC subunits exhibit patterns of variability consistent with selection for new or altered function. We propose that these latter subunits participate in regulatory interactions important for differential usage of poly(A) sites.

## 1 Introduction

Messenger RNA polyadenylation process is an essential step for gene expression and regulation in eukaryotes (Edmonds, 2002). This process is mediated by a complex (the polyadenylation complex, or PAC) that consists of between 15 and 20 subunits (Boreikaitė and Passmore, 2023). These subunits function in the context of a set of subcomplexes, scaffolds, and enzymes - Cleavage and Polyadenylation Specificity Factor (CPSF), Cleavage stimulatory Factor (CstF), Cleavage Factor I (CFIm) and II (CFIIm), FIP1, symplekin, RBBP6, Poly(A) Polymerase (PAP), and Poly(A) Binding Protein-Nuclear (PABN). In mammals, CPSF recognizes the conserved AAUAAA hexamer cis-element and cleaves the pre-mRNA at the polyadenylation site (Zhao et al., 1999; Chan et al., 2014; Schonemann et al., 2014; Clerici et al., 2018; Zhang et al., 2020). CstF recognizes sequences downstream of the mammalian poly(A) site and plays important roles in regulating alternative polyadenylation (APA) and in mediating between transcription and DNA repair (Pérez Cañadillas and Varani, 2003; Mirkin et al., 2008; Grozdanov et al., 2018; Yang et al., 2018; Zhang et al., 2020). CFIm recognizes the UGUA upstream cis-element and influences alternative poly(A) site selection, mRNA export and mRNA splicing (Martin et al., 2012; Zhu et al., 2018; Ghosh et al., 2022). Cleavage factor II (CFIIm) contributes to the recognition of cleavage/polyadenylation substrates through interaction with G-rich far-downstream sequence elements (Schäfer et al., 2018). CFIIm also plays roles in transcription termination (Sadowski et al., 2003; Zhang et al., 2005; West and Proudfoot, 2008; Kamieniarz-Gdula et al., 2019). FIP1 is a scaffold that links PAP with other parts of the PAC (Helmling et al., 2001; Kaufmann et al., 2004; Zhang et al., 2020; Muckenfuss et al., 2022). Symplekin and RBBP6 are additional scaffolds that coordinate the subcomplexes and enzymes in the course of the reaction (Ghazy et al., 2009; Kennedy et al., 2009; Xiang et al., 2010; Ruepp et al., 2011; Di Giammartino et al., 2014; Zhang et al., 2020; Rodríguez-Molina et al., 2022; Schmidt et al., 2022). PAP is the nucleotidyltransferase that adds the poly(A) tract to the 3’ end of the cleaved pre-mRNA (Bard et al., 2000). In mammals, PABN regulates the length of poly(A) tail (Kerwitz et al., 2003; Kuhn and Wahle, 2004; Kühn et al., 2009).

With the possible exception of RBBP6 (discussed below), higher plants possess orthologs for the suite of core subunits of the mammalian and yeast PACs (Hunt et al., 2012). In plants, various PAC subunits have been implicated in important aspects of plant growth and development. CPSF30 is important in linking environmental signals and poly(A) regulation (Bruggeman et al., 2014; Hunt, 2014; Chakrabarti and Hunt, 2015). Both CPSF30 and FIP1 proteins participate in nitrate signaling and regulation (Li et al., 2017; Tellez-Robledo et al., 2019; Hou et al., 2021). In addition, FIP1 is important for plant response to stress and root development (Tellez-Robledo et al., 2019) and for seed dormancy (Li et al., 2023). CstF77 and CstF64 have been linked with the control of flowering time (Liu et al., 2010) and with responses to auxin (Zeng et al., 2019). One of the two *Arabidopsis* CFIm25 orthologs is important for maintaining the 3’ UTR length in *Arabidopsis*, and mutation of this ortholog causes abnormal phenotypes (Zhang et al., 2022). CPSF73 plays roles in reproductive development in *Arabidopsis* (Xu et al., 2006). CPSF100 has functions in embryogenesis, seed production and root development (Lin et al., 2017). One Pcf11 ortholog, PCFS4, plays roles in the control of flowering time (Xing et al., 2008b). CLPS3 functions in embryo development (Xing et al., 2008a). Different PAP orthologs have been linked with the control of flowering time, defense responses, and aspects of gamete development and function (Vi et al., 2013; Trost et al., 2014; Kappel et al., 2015; Czesnick and Lenhard, 2016; Zhang et al., 2019; Ramming et al., 2023).

In plants, APA has been linked to numerous biological processes. For example, the choice of proximal and distal poly(A) site choice of transcripts encoded by the FCA gene, controlled by the core PAC subunit FY, determines the expression of FCA, a regulator of flowering time (Simpson et al., 2003). FY and FCA moreover cooperate to determine the usage of distal or proximal poly(A) sites associated with antisense transcripts that in turn regulate expression of FLC, a central regulator of flowering time in *Arabidopsis* (Whittaker and Dean, 2017). Usage of the poly(A) sites associated with antisense FLC transcripts is also linked with CstF77 and CstF64 (Simpson et al., 2003; Henderson et al., 2005; Liu et al., 2010; Whittaker and Dean, 2017). On a more global basis, poly(A) site choice varies genome-wide at different developmental stages in rice and *Arabidopsis* (Shen et al., 2011; Fu et al., 2016; Zhou et al., 2019). A large number of genes undergo APA in response to abiotic and biotic stress in sorghum (Chakrabarti et al., 2020), rice (Fu et al., 2016; Ye et al., 2019), *Populus trichocarpa* (Yan et al., 2021) and *Arabidopsis* (Hunt, 2014; de Lorenzo et al., 2017; Ma et al., 2022). Several plant PAC subunits have been implicated in the regulation of APA, including CstF77 (Zeng et al., 2019; Kim et al., 2023), CPSF30 (Liu et al., 2014), FIP1 (Tellez-Robledo et al., 2019), and FY (Yu et al., 2019).

While the impact of APA in plants is clear, much remains to be learned regarding the mechanisms that connect the PAC with environmental and developmental cues. Chief among the outstanding questions is that regarding the interactions of different PAC subunits with the larger gene regulatory network. One approach towards a better understanding of enzymes, complexes, and processes involves the assessment of naturally-occurring variability in the respective proteins (Alonso-Blanco et al., 2016; Hamm et al., 2019; Kadirjan-Kalbach et al., 2019; Zan and Carlborg, 2019). In this study, we compile and assess naturally-occurring variants in the subunits of the *Arabidopsis* PAC. Our results reinforce other studies that indicate essential roles for many core PAC subunits. In addition, they suggest that a subset of PAC subunits may be subject to diversifying selection, possibly indicative of functional specialization and roles in regulatory processes. Our results identify several genes as probable pseudogenes, thus tightening the focus of PAC subunits in *Arabidopsis* and answering questions about their absence in most other plants. Finally, we find that two evolutionarily-conserved PAC subunits, CstF50 and PAPS3, may not be essential in *Arabidopsis*, raising questions about their widespread conservation and possibilities about their roles in the PAC and in APA.

## 2 Methods

### 2.1 Plant growth and characterization

Four *Arabidopsis* strains (CS76822, CS76769, CS77397, CS7884) were ordered from the ABRC Stock center. Seeds were sown in soil, and grown in a temperature-controlled growth room at 22°C with a 16/8 hr light/dark cycle. After 20 days growth, leaves were collected and DNA was isolated using Plant DNAzol (Life Technologies) following the manufacturer’s instructions. The respective regions of interest were amplified by PCR using the primers listed in **Supplementary File 7**. PCR reactions consisted of: 0.25 ul Phire Hot Start II DNA Polymerase, 5 ul 5X phire reaction buffer, 2.5 ul 2.5 mM dNTP, 1 ul 10 uM forward primer, 1 ul 10 uM reverse primer, 1 ul of extracted DNA (concentration range 200-400 ng/ul), and 14.25 ul water. The cycle temperatures and durations were 95°C for 15 seconds, 55°C for 15 seconds, and 72°C for 30 seconds. PCRs were run for 25 cycles. PCR products were gel-purified using QIAquick Gel Extraction Kit as described in the user’s manual. PCR products were sequenced by Eurofins Genomics; primers for sequencing reactions are indicated in **Supplementary File 7**. Sequencing results were aligned to the Col-0 reference sequences and displayed to confirm the homozygous nature of mutations; bioinformatics was conducted using various tools in the CLC Genomics Workbench package. After sampling for DNA, plants were grown until flowering, and then photographed.

### 2.2 Data collection and analyses

SNPs and variants that affect the protein coding regions (and not non-coding parts of genes such as promoters, untranslated regions, and introns) for the 31 genes that encode probable PAC subunit orthologs were downloaded from the *Arabidopsis* 1001 Genomes website using Polymorph 1001 tools; the list of genes is given in **Table 1**. The PCFS2 and SYM annotations in the *Arabidopsis* 1001 database were from an outdated annotation and were accordingly updated prior to data downloading. Specifically, PCFS2 was “formed” by merging the AT2G36485 and AT2G36480 annotations, and SYM by merging AT1G27590 and AT1G27595. *Arabidopsis* orthologs of the human RBBP6 were identified using BLASTP with the human RBBP6 as a query; this yielded two possible orthologs (denoted Mpe1 and PQT3 in **Table 1** and elsewhere in this study).

**Table 1:** A list of *Arabidopsis* polyadenylation complex subunits.

transcript id	gene name
AT1G13190.1	CFIm68-1
AT5G55670.1	CFIm68-2
AT4G29820.1	CFIS1
AT4G25550.1	CFIS2
AT3G04680.2	CLPS3
AT5G39930.1	CLPS5
AT5G23880.1	CPSF100
AT5G51660.1	CPSF160
AT1G30460.1	CPSF30
AT1G61010.3	CPSF73
AT5G60940.1	CSTF50
AT1G71800.1	CSTF64
AT1G17760.1	CSTF77
AT5G01400.1	ESP4
AT3G66652.1	FIPS3
AT5G58040.1	FIPS5
AT5G13480.1	FY
AT5G51120.2	PABN1
AT5G65260.1	PABN2
AT5G10350.1	PABN3
AT1G17980.1	PAPS1
AT2G25850.1	PAPS2
AT3G06560.1	PAPS3
AT4G32850.10	PAPS4
AT1G66500.1	PCFS1
AT2G36485.1 + AT2G36480.3	PCFS2
AT4G04885.1	PCFS4
AT5G43620.1	PCFS5
AT1G27590.1 + AT1G27595.1	SYM
AT4G17410	PQT3
AT5G47430	Mpe1

**Figure 1:**
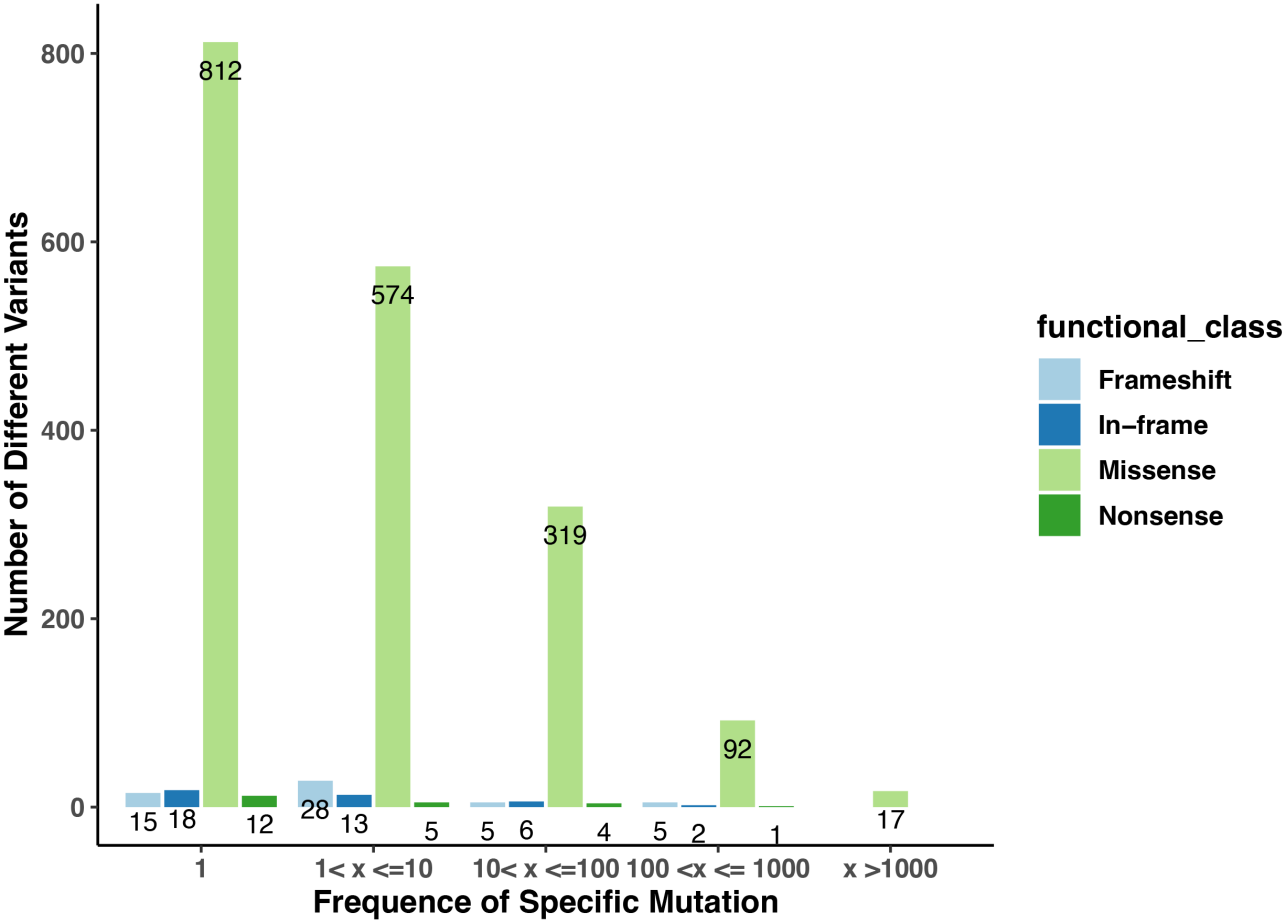
The numbers of variants in the collection of *Arabidopsis* ecotypes in the *Arabidopsis* 1001 Genomes database that affect genes encoding PAC subunits. The numbers on the plot indicate the counts of variants with frequencies falling within the specified ranges. The x-axis displays the different ranges, while the y-axis represents the counts of missense, nonsense, frameshift, and in-frame (insertion or deletion) mutations observed in each category. Distinct colors are used to denote four different functional classes.

**Figure 2:**
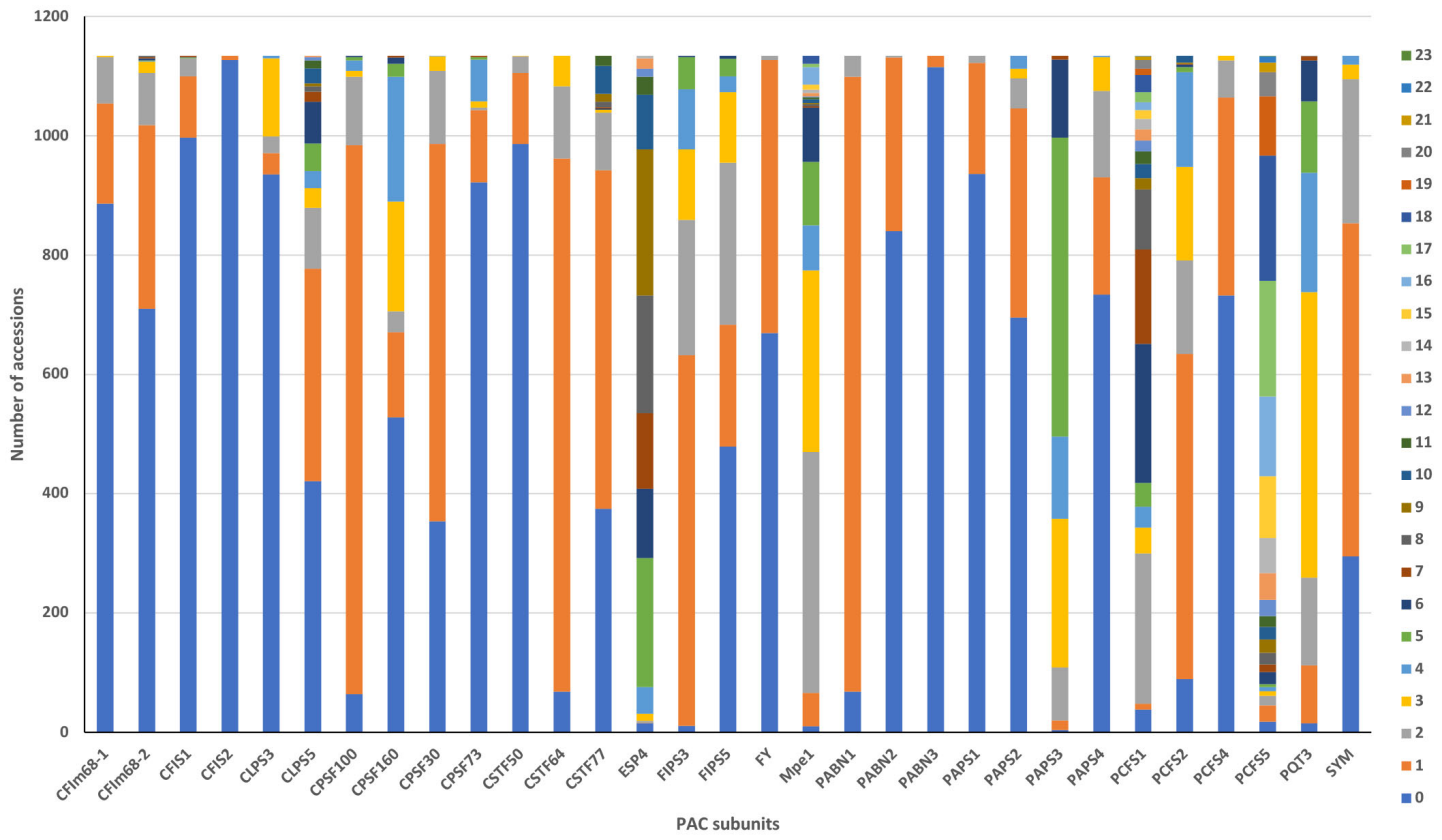
Summary of the numbers of ecotypes that possess missense changes in genes encoding PAC subunits. Colors denote the numbers of variants found in each accession, with the code indicated in the legend. The x axis displays the name of the PAC subunits, and the y axis represents the number of accessions bearing the indicated numbers of changes.

The missense, silent mutations, nonsense mutations, and indels for each gene were tabulated and assembled into **Supplementary File 1**. These data were used to evaluate various features as described in the text. R studio software (data.table, dplyr, ggplot2 packages) was used to calculated the frequency for each PAC and draw **Figures 1** and **2**.

Analyses of synonymous and nonsynonymous substitutions were conducted using the Visualizing Variation (ViVa) analysis package (Hamm et al., 2019) run in R. This package extracts and compiles sequence variation information from the *Arabidopsis* 1001 Genomes database; included in the compilation are calculations of ratios of collective non-synonymous to synonymous diversity (π_N_/π_S_) for each protein-coding region. Details of the use of this package and of the π_N_/π_S_ calculations may be found in Hamm et al. (Hamm et al., 2019).

To determine the expression of PAPS3-like genes in different plant species, PAPS-like genes in a select set of plant species were identified by BLASTP using the *Arabidopsis* PAPS3 protein sequence as a query; for this, plant species were chosen based on their presence in the EVOREPRO database (https://evorepro.sbs.ntu.edu.sg). These genes were used as queries to extract expression information in different tissues. Expression results were displayed after normalizing each sample such that the lowest expression level was equal to 1.

### 2.3 Genome re-assemblies

To reassemble and analyze the genomes of selected accessions, the respective short reads were downloaded from SRA (SRP056687). SRA accessions used in this study were SRR1946375 (for *Arabidopsis* accession 9812), SRR1945601 (accession 5984), SRR1946283 (accession 9705), and SRR1946188 (accession 9596). *De novo* assembly for each set of reads was done using the *De Novo* Assembly tool in the CLC Genomics Workbench (versions 20-23 were used in the course of this research), using the default parameters (Automatic Word Size, Automatic Bubble Size, minimum contig length = 200 bp). The results of each assembly are given in **Supplementary Files 4** and **5**. The sets of contigs for each sample were used to create blast databases, which were then used in TBLASTN searches. TBLASTN searches were conducted using the respective Col-0 amino acid sequences.

### 2.4 PCFS1, PCFS5, and CLPS5 poly(A) site profiles

To confirm APA profiles for the PCFS1, PCFS5, and CLPS5 genes, 3’ end profiling (Poly(A) Tag Sequences, or PATSeq) datasets from four published studies were downloaded and analyzed; for this, only Col-0 control data were used. These datasets were from studies of *Arabidopsis* mutants affected in various PAC subunits (CstF77 and CstF64 (Zeng et al., 2019), FY (Yu et al., 2019) and CPSF30 (Hou et al., 2021) and from a characterization of poly(A) site choice in plants subjected to hypoxic conditions (de Lorenzo et al., 2017). SRA accessions are given in **Supplementary File 8**. PATSeq reads were mapped to the *Arabidopsis* genome (TAIR10 reference) using the read mapping tool in the CLC Genomics Workbench package. For this, genomic regions adjacent to tracts of 8 or more A’s were masked. The mapping parameters used were: Match score = 1, Mismatch cost = 2, Cost of insertions and deletions = Linear gap cost, Insertion cost = 3, Deletion cost = 3, Length fraction = 0.9, Similarity fraction = 0.9, Global alignment = No, Non-specific match handling = Map randomly, Execution mode = Standard, Minimum seed length = 15.

An additional Col-0 PATSeq dataset was generated for this study. Col-0 seeds were surface-sterilized by incubating in 70% ethanol for 1 min, followed by treatment with 10% bleach for 10 min, and then rinsed with distilled water five times. After the sterilization, seeds were suspended in a 0.1% agar solution and sowed onto ½ MS medium with 1% (w/v) sucrose, 0.8% (w/v) agar. Following stratification for 2 d in the dark at 4°C, plates were transferred to a growth chamber and incubated under long-day conditions (16 h light/8 h dark) at 22°C for 3 weeks. At this time, whole seedlings were removed, frozen in liquid nitrogen, the frozen tissue ground in a mortar and pestle, and RNA extracted using TRIzol RNA Isolation Reagents (Life Technologies) as recommended by the manufacturer. Short read sequencing libraries that query the mRNA-poly(A) junction (PAT-Seq libraries) were prepared as described previously (Ma et al., 2014; Pati et al., 2015). 1-3 µg of total RNA brought to 50 µL in 1X NEB RNA Fragmentation Buffer and incubated at 95°C for 2 min. Fragmented RNAs bearing poly(A) tracts were purified using the NEB Poly(A) RNA Isolation Kit and eluted in a final volume of 14 µL. The entire sample was then used as a template for SMART cDNA synthesis using Smartscribe (Takara); for this, the poly(A)-enriched RNA was incubated in 25 µL of 1X RT buffer (prepared from the 5X stock provided by the manufacturer) containing 1 mM dNTPs, 1 mM DTT, 4 µM RT primer (see **Supplementary File 7**), and 1 µL of enzyme as supplied by the manufacturer. After 30 min at 42°C, 100 pmol of the strand-switching primer (SMART7.5; see **Supplementary File 7**) and an additional 1 µL of enzyme were added and reactions incubated for an additional 30 min at 42°C. After a subsequent incubation at 70°C for 5 min, 16.25 µL of SPRI beads (HighPrep PCR, Magbio Genomics, Inc.) was added, the solution completely mixed, and incubated for 8 min at room temperature. Beads were collected on a magnetic stand, washed twice with 100 µL 80% ethanol, air-dried for 5 min, and bound cDNA eluted with 25 µL water. 1 µL of the eluted cDNA was used for a limited PCR amplification using Phire Hot Start II DNA Polymerase (Thermo Fisher) and PE-PCR1 and PE-PCR2 primers. The cycle temperatures and durations were 95°C for 15 seconds, 60 °C for 15 seconds, and 72°C for 60 seconds. Reactions were run for 15 cycles. PCR products were separated on 1.5% agarose gels and products ranging between 300 and 500 bp excised and purified using a Qiagen gel purification kit. The gel-purified fragments were re-amplified using the same PCR conditions; PCR products at this point were purified using SPRI beads as described above. This final library was quantified using a Qubit and submitted for sequencing on an Illumina HiSeq2500 instrument at the University of Kentucky HealthCare Genomics Core Laboratory. PATSeq reads were analyzed using the pipeline described in the preceding paragraph and elsewhere (Thomas et al., 2012; Thomas, 2015; de Lorenzo et al., 2017). These sequencing data are available under Bioproject PRJNA1023006.

## 3 Results

### 3.1 Naturally-occurring sequence variation affecting the *Arabidopsis* polyadenylation complex – an overview

To study possible variability in the *Arabidopsis* PAC, genetic variants in a large collection of *Arabidopsis* accessions (Alonso-Blanco et al., 2016) that affect different subunits of the PAC were compiled and tabulated. The PAC subunits, notations used in this report, and corresponding gene identifiers are listed in **Table 1**. Earlier compilations of plant PAC subunits (Hunt et al., 2008; Hunt et al., 2012) lacked mention of possible orthologs of RBBP6/Mpe1, a scaffold protein that coordinates processing and polyadenylation activities of the mammalian and yeast complexes (Di Giammartino et al., 2014; Lee and Moore, 2014; Hill et al., 2019; Lee et al., 2020; Boreikaite et al., 2022; Rodríguez-Molina et al., 2022; Schmidt et al., 2022). For the sake of completeness, *Arabidopsis* RBBP6/Mpe1 orthologs were identified with BLASTP; this analysis yielded two possible counterparts, encoded by AT4G17410 and AT5G47430 (**Supplementary Figure 1**). One of these proteins (AT5G47430) is present in nuclear complexes containing CstF77 (Antosz et al., 2017); for this reason, these two proteins are included in this compilation and analysis. To facilitate subsequent analyses, the gene designations for these subunits that are in the ViVa (Hamm et al., 2019) database were retained.

1814 non-redundant missense SNPs were identified in genes encoding PAC subunits. Of these, 55% (1002/1814) were observed in at least two accessions, with 17 variants found in more than 1000 accessions (**Figure 1**). These 17 variants affected 10 of the 31 genes of interest (**Table 2**). Additionally, 22 nonsense mutations (affecting 9 of the 31 genes) and 53 frameshift variants (affecting 13 of the 31 genes) were found (**Figure 1**; **Tables 3**, **4**; **Supplementary File 1**). Notably, one of the nonsense mutations, affecting the CLPS5 gene, occurred in almost half of the accessions (**Table 3**; **Supplementary File 1**). Specific frameshift mutations in three genes (PCFS5, PQT3, FIPS3) were observed in more than 100 accessions (**Table 4**). Seven genes with frameshift variants were also among those with nonsense variants (**Tables 3**, **4**). These findings indicate that the 31 genes of interest exhibit distinct amino acid sequences in *Arabidopsis* strains, and some of them may lose function in specific strains.

**Table 2:** Missense mutations seen in more than 1000 accessions.

Gene_Name	Wild_Type	Position	SNP	Frequency
PCFS1	Asp	364	Ala	1077
PCFS1	Leu	217	Ser	1042
CSTF64	Phe	363	Tyr	1066
PAPS3	Ile	180	Ser	1100
PAPS3	Thr	297	Asn	1013
PAPS3	Leu	312	Arg	1098
FIPS3	Tyr	273	Asp	1111
ESP4	Asp	1398	Glu	1096
ESP4	Met	791	Lys	1096
ESP4	Gly	585	Val	1062
CPSF100	Ile	441	Val	1066
PCFS5	Val	115	Gly	1009
PCFS5	Asn	215	Ser	1013
PABN1	Lys	89	Glu	1065
PQT3	Ser	715	Pro	1050
Mpe1	Pro	809	Ser	1048
Mpe1	Thr	693	Pro	1044

**Table 3:** Nonsense Mutations in genes encoding PAC subunits.

Gene_Name	Wild_Type	Position	Frequency
PCFS1	Gly	5	1
PAPS2	Arg	307	2
PAPS3	Trp	54	2
PAPS3	Glu	198	2
PAPS3	Leu	209	1
PAPS3	Tyr	492	13
PABN3	Glu	33	1
CLPS5	Trp	421	1
CLPS5	Gln	259	1
CLPS5	Arg	209	1
CLPS5	Arg	98	21
CLPS5	Gln	97	547
CLPS5	Gln	95	1
CLPS5	Arg	18	18
PCFS5	Tyr	83	9
PCFS5	Ser	95	1
PCFS5	Leu	99	1
PCFS5	Arg	281	1
PCFS5	Gln	284	3
FIPS5	Arg	1186	28
CSTF50	Arg	212	1
PQT3	Gln	453	1

**Table 4:** Frameshift Variants in genes encoding PAC subunits.

gene_name	sequence_change	Frequency
CFIS1	p.Ser14_Asp15fs/c.42_43insC	6
CFIS1	p.Ser14_Asp15fs/c.40_41insAT	4
CLPS5	p.Tyr401fs/c.1202_1203delAT	5
CLPS5	p.Ile399fs/c.1195delA	12
CLPS5	p.Arg398_Ile399fs/c.1194_1195ins	1
CLPS5	p.Val322_Lys323fs/c.966_967insT	1
CLPS5	p.Gly238fs/c.713delG	12
CLPS5	p.Val235fs/c.705_708delTCGT	1
CLPS5	p.Lys182_Ala183fs/c.545_546insA	14
CLPS5	p.Phe147_Val148fs/c.441_442insAA	4
CLPS5	p.Lys140fs/c.420_432delAGATGGTTG	1
CLPS5	p.Ser79fs/c.235delT	1
CLPS5	p.Ala36fs/c.106delG	1
CLPS5	p.Glu16_Leu17fs/c.46_47insCG	10
CLPS5	p.Gly3fs/c.9delT	3
CSTF50	p.Ser120fs/c.358delT	2
CSTF50	p.Val249fs/c.746_753delTAAACACA	1
ESP4	p.Gly585fs/c.1754_1755delGG	2
ESP4	p.Asp584fs/c.1751delA	3
FIPS3	c.2993_2994insA	807
PABN1	p.Glu124fs/c.371delA	1
PAPS2	p.Asn697_Glu698fs/c.2090_2091ins	6
PAPS3	p.Asp36fs/c.108delT	2
PAPS3	p.Ile76_Leu77fs/c.226_227insA	3
PAPS3	p.Asp108_Phe109fs/c.324_325insT	2
PAPS3	p.Asn221_Gly222fs/c.661_662insA	3
PAPS3	p.Phe430fs/c.1290delC	1
PAPS3	p.Leu441fs/c.1321_1325delCTTGT	3
PAPS3	p.Lys460fs/c.1378delA	20
PAPS4	p.His739fs/c.2217_2218delTG	2
PCFS1	p.Thr168fs/c.504_505delTC	1
PCFS1	p.Ser142fs/c.424delT	9
PCFS1	p.Gly118_Asn119fs/c.352_353insA	2
PCFS1	p.Ser80fs/c.240_241delTC	1
PCFS2	p.Thr876fs/c.2626delA	1
PCFS2	p.His866fs/c.2597delA	9
PCFS2	p.Ser141_Cys142fs/c.421_422insT	1
PCFS5	p.Asp89fs/c.267_268delTG	15
PCFS5	p.Ala98_Leu99fs/c.294_295insT	3
PCFS5	p.Asn118fs/c.354delC	5
PCFS5	p.Asn175fs/c.525_526delCA	129
PCFS5	p.Met176fs/c.528_534delGGTTTCA	246
PCFS5	p.Asn226fs/c.678delT	4
PCFS5	p.Ile238fs/c.713delT	7
PCFS5	p.Gln331fs/c.993_994delAC	3
PCFS5	p.Val334_Pro335fs/c.1000_1001ins	8
PCFS5	p.Ala344fs/c.1031delC	6
PCFS5	p.Leu345fs/c.1033delT	5
PQT3	p.Trp418_Ala419fs/c.1252_1253ins	430
PQT3	p.Trp418_Ala419fs/c.1253_1254ins	311
PQT3	p.Glu664fs/c.1990_1991delGA	1
PQT3	p.Arg665fs/c.1995delT	1
PAPS3	p.Cys507fs/c.1519_1528delTGTTAGG	10

All 1134 of the strains in the 1001 Genomes collection possessed variations (compared to the Col-0 reference) that affect the amino acid sequences of PAC subunits. The numbers of such variants in specific strains ranged from 9 (in Lan-0) to 106 (in IP-Vis-0) (**Supplementary File 2**). Many accessions had multiple missense variants in different PAC subunits; the range of variants in particular subunits ranged from 1 to 23 (**Figure 2**, **Supplementary File 2**). For 9 genes, the Col-0 reference sequence was the one seen in >70% of accessions (**Figure 2**; **Supplementary File 2**). For another 10 genes, either the Col-0 reference or a single amino acid substitution was seen in >70% of accessions (**Figure 2**; **Supplementary File 2**). For the remaining genes, the range and frequency of substitutions was broad.

A subset of genes showed a striking extent of variation, indicated by numerous accessions with multiple substitutions in each gene (**Figure 2**; **Supplementary File 2**); this subset consisted of the CSTF77, MPE1, CLPS5, ESP4, PAPS3, PCFS1, and PCFS5 genes. The scope of variation in PCFS5 was especially striking, with 86% of the accessions having more than 10 missense substitutions in this gene (**Figure 2**; **Supplementary File 2**).

### 3.2 Purifying and diversifying selection in *Arabidopsis* genes encoding PAC subunits

To further assess the variation affecting PAC subunits, the ratio of collective non-synonymous to synonymous diversity (*π_N_/π_S_*) for each gene was determined using the tool provided in the ViVa package (Hamm et al., 2019). Analogous to determinations of the rates of non-synonymous and synonymous substitutions, the *π_N_/π_S_* ratio derived from ViVa provides information about the overall conservation of amino acid sequence and consequently of functional diversity in the collection (Hughes, 1999; Hughes et al., 2000). Among the information is that concerning the tendencies towards purifying or diversifying evolution for specific genes. This tool has been shown useful in lending new and interesting insights into the nuclear auxin signaling pathway, identifying ARF members subjected to differing extents of purifying or diversifying evolution (Hamm et al., 2019). Demarcation of PAC subunits along these lines could be informative. Accordingly, the PAC-associated genes listed in **Table 1** were analyzed using this tool.

**Figure 3:**
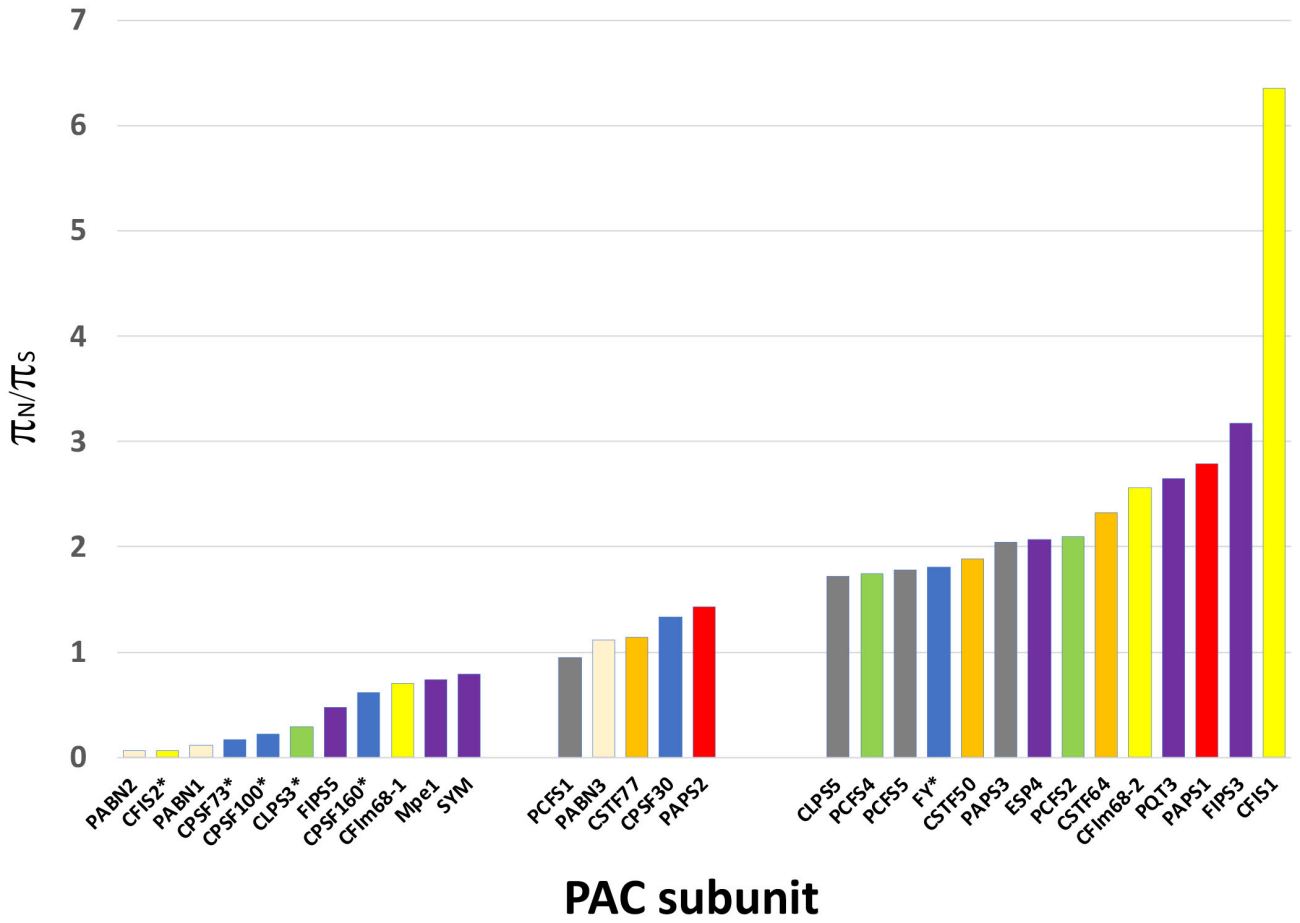
Results of the Visualizing Variation (ViVa) analysis. The π_N_/π_S_ ratio for each gene is plotted as shown. Genes are grouped according to their π_N_/π_S_ values - π_N_/π_S_ ratios less than 0.8, ratios between 0.8 and 1.5, and with ratios greater than 1.5. The y axis represents the π_N_/π_S_ value. Stars indicate genes that are essential. Data for this plot is provided in **Supplementary File 3**.

**Figure 4:**
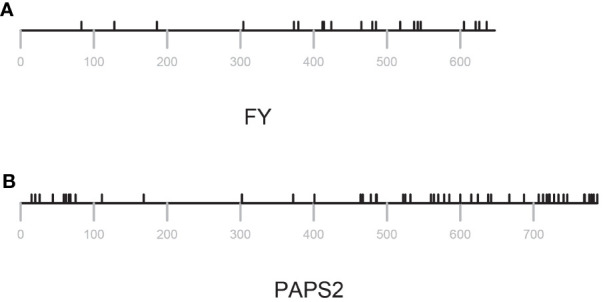
Distribution of missense changes in FY and PAPS2. Locations of variants are indicated by black tics above the lines that represent the amino acid sequence. Position numberings are shown with gray tics beneath the sequence representation. **(A)** FY **(B)** PAPS2.

As shown in **Figure 3**, the range of *π_N_/π_S_* ratios in PAC-associated genes ranged from 0.064 to 6.4. PAC-associated genes could be loosely divided into three groups (**Figure 3**; **Supplementary File 3**) – those with *π_N_/π_S_* ratios less than 0.8, those with ratios between 0.8 and 1.5, and with ratios greater than 1.5 (**Figure 3**; **Supplementary File 3**). The various PAC subcomplexes (CPSF, CstF, etc.) and other functional groups (scaffold proteins, poly(A) polymerases, PABNs) have members with low and high *π_N_/π_S_* ratios (**Figure 3**). All but one of the known essential PAC subunits have ratios less than 0.8. The exception (FY) has a ratio greater than 1.5 (**Figure 3**; **Supplementary File 3**). For this protein, the majority of missense mutations affect the C-terminus of the protein (**Figure 4A**; **Supplementary Figure 2A**).

Many PAC subunits are encoded by more than one gene. For several of these – PABN, CFIS, CFIm-68, FIPS, PAPS, and symplekin (SYM/ESP4) – one or more genes had ratios greater than 1.5 and others had *π_N_/π_S_* ratios less than 0.8 (**Figure 3**). For some pairs, the contrast between genes was striking. Specifically, for CFIS, CFIm68, FIPS, and SYM, one of the respective duplicate genes (CFIS2, CFIm68-1, FIPS5, and SYM) had low *π_N_/π_S_* ratios, while the other member of each duplicate set had ratios greater than 2 (**Figure 3**).

Low *π_N_/π_S_* may be reflective of purifying evolution and conservation of sequence and function, while high *π_N_/π_S_* ratios perhaps suggestive of a trend towards diversification. *π_N_/π_S_* ratios nearer 1 might reflect a more neutral mode of evolution, and thus of a protein not subject to strong selective pressures. Five of the set of genes associated with the PAC has this feature – PCFS1, PABN3, CstF77, CPSF30, and PAPS2. One of these, PCFS1, is a probable pseudogene (see the following). Two, CstF77 and CPSF30, are single-copy genes whose proteins have core functions in the PAC. However, these two genes are also non-essential (Zeng et al., 2019), a feature that may be related to their possible neutral evolution. PAPS2 is one of three nuclear PAPS isoforms in *Arabidopsis* and other plants. The *Arabidopsis* isoforms show a degree of functional specialization that may be attributed to the divergent C-termini of the proteins. The missense variants in these genes are largely clustered near the 3’ ends of the respective coding regions (**Figure 4B**; **Supplementary Figure 2B**).

### 3.3 The distributions of nonsense and frameshift variants provide novel insights into the functions of several PAC-associated genes

Included in the variability that affects genes encoding PAC subunits are 22 nonsense mutations and 53 frameshift variants (insertions or deletions). These variants affect 13 genes (**Tables 3**, **4**). In several of these genes, the changes fall near the C-termini of the corresponding coding regions, and likely do not affect the functionality of the respective gene (**Supplementary Figure 3**). Others, however, are predicted to have a large impact on gene functionality, due to severe truncations of the respective protein-coding regions (**Supplementary Figure 3**). Several of these affect members of small gene families; included in this set are genes encoding PABN1, PABN3, PQT3, CFIS1, ESP4, FIPS3, and PCFS2. Still others affect genes that are not members of families, or are unique to the *Arabidopsis* lineage. These latter genes – encoding CstF50, PAPS3, PCFS1, PCFS5, and CLPS5 – are interesting and provocative and are discussed in the following subsections.

#### 3.3.1 The *Arabidopsis* CstF50 gene is not required for growth and development

**Figure 5:**
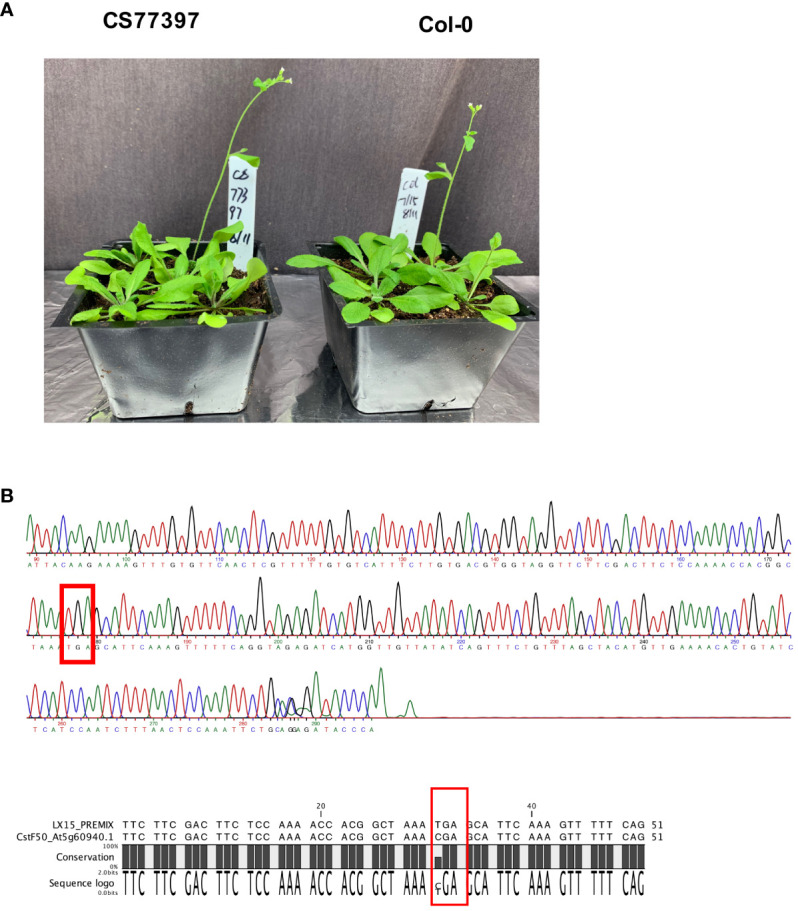
Characterization of *Arabidopsis* line CS77397. **(A)** Photograph of soil-grown CS77397 and Col-0 plants. **(B)** Sanger sequencing trace of the region encompassing the nonsense variant seen in CS77397. The location of the change is highlighted with a red box. The alignment beneath the trace shows a comparison of the Col-0 and CS77397 sequences, confirming the alteration that is noted in the *Arabidopsis* 1001 Genomes database.

One *Arabidopsis* accession (CS77397) had a premature stop codon within the CstF50 gene, and three others (CS78771, CS78772 and CS76987) had frameshift variants (**Tables 3**, **4**; **Supplementary File 1**). The locations of these changes (**Supplementary Figure 3**) imply an inactivation of this gene in the respective accessions. This was unexpected, as CstF50 is essential in mammals and yeast and the *Arabidopsis* CstF50 gene (At5g60940) is a single copy gene. To confirm these suggestions, the CS77397 line was further characterized. Soil-grown plants had typical appearances, flowering behaviors, and fertility (**Figure 5A**; **Supplementary Figure 4**). The DNA sequence of the affected site was determined after PCR amplification and cloning. The results confirmed the presence of the mutation in a homozygous state (**Figure 5B**), with no suggestion of an additional copy of the gene that might encode a wild-type copy of the gene. To test the possibility that the CstF50 gene in this line has been duplicated, the raw re-sequencing data for this accession were re-assembled and the assembly searched to identify all contigs that may possess CstF50-related sequences. This exercise yielded a single contig that could encode a polypeptide with substantial identity to CstF50 (**Supplementary File 4**). While this experiment does not rule out large-scale (chromosome-sized) structural variants, it does indicate that there are no additional CstF50 genes that lack a stop codon in this accession (CS77397). These results indicate that CstF50 is not essential for *Arabidopsis* growth and development.

#### 3.3.2 Three genes that encode putative CFIIm subunits are pseudogenes

The canonical mammalian factor CFIIm consists of two subunits, Pcf11 and Clp1. *Arabidopsis* possesses four possible Pcf11-encoding genes and two Clp1 genes [termed as PCFS and CLPS in this report, as suggested by others (Hunt et al., 2008; Hunt et al., 2012)]. Six nonsense and 18 frameshift mutations affecting three of the PCFS genes were found in the collection of *Arabidopsis* accessions (**Tables 3**, **4**; **Supplementary Figure 3**). These mutations occur in a large number of accessions. Specifically, fifteen accessions contain premature termination codons in PCFS5 and one accession has a premature termination codon in PCFS1 (**Table 3**). However, no accessions possess premature termination codons in both PCFS1 and PCFS5. Numerous other accessions possess frameshifts in either PCFS1 or PCFS5 (but not both; **Table 4**; **Supplementary Figure 3**).

**Figure 6:**
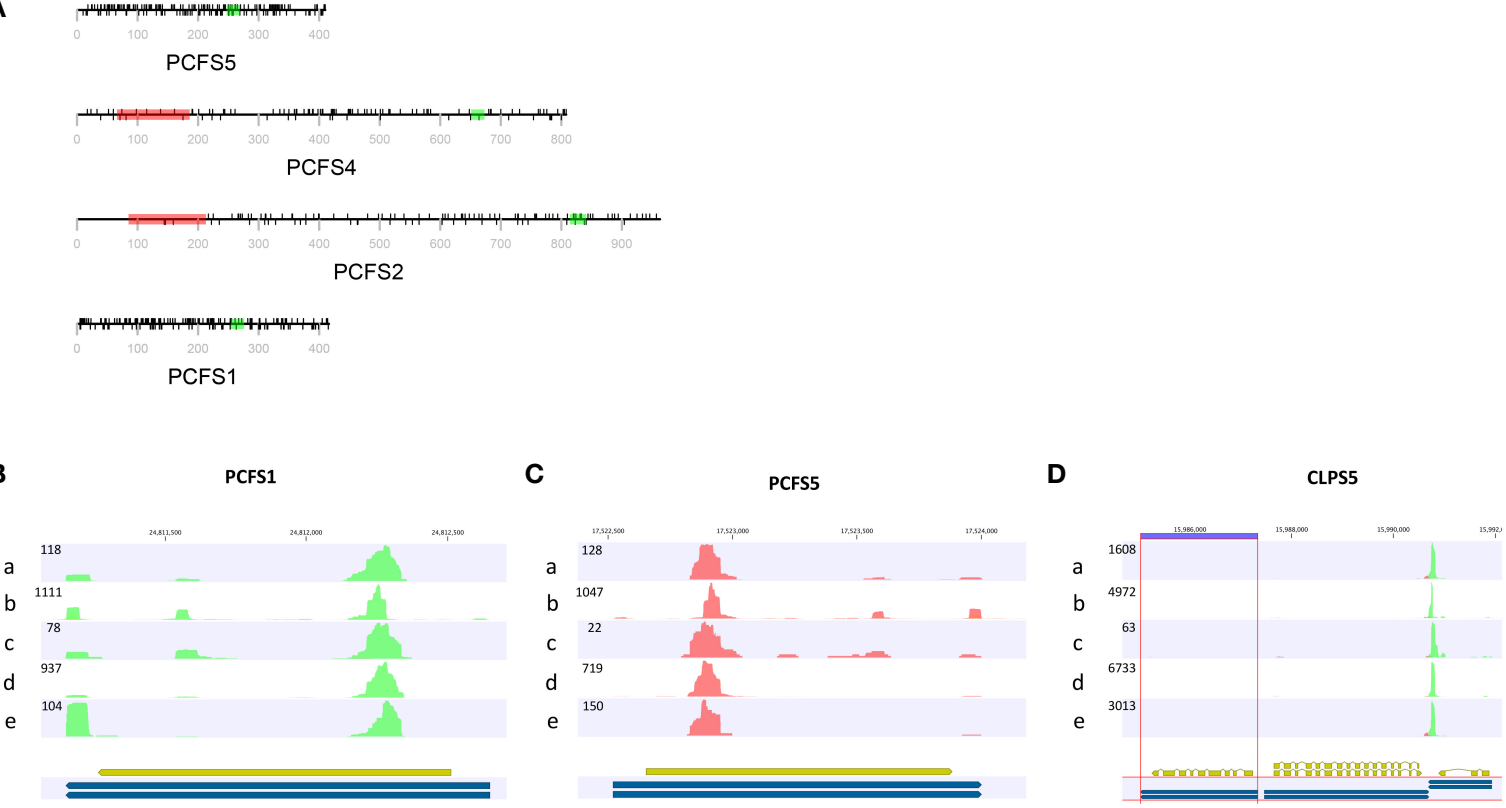
Feature of genes encoding CFIIm subunits. **(A)** The domain structures of *Arabidopsis* Pcf11 orthologs (PCFS). Red rectangles in represent the CID domain, green rectangles the zinc finger motif. The black lines above the protein representation are missense mutations, and those below are silent mutations. The grey line and text represent the amino acid positions and overall polypeptide length. **(B–D)** PATSeq mapping tracks showing the 3’ ends (as well as overall expression levels) of the PCFS1, PCSF5, and CLPS5 genes. Tracks a-d were derived from a re-analysis of four different PATSeq experiments as described in Methods and in **Supplementary File 8**. Track e was generated in this study as described in Methods. Blue and gold bars beneath each track show the respective gene annotations. Numbers above each track show the respective chromosome positions. Numbers at the upper left of each track denote the sizes (mapped reads) of the largest peaks. Green reads indicate those that read right-to-left (5’-3’) and red reads those that read left-to-right (5’-3’). Poly(A) sites are the left-most (for green reads) or right-most (for red reads) parts of each peak.

These two genes are distinctive in other ways. As noted above (**Figure 3**; **Supplementary File 1**), there is extensive missense variation in these two genes (109 missense found in PCSF1, and 123 missense mutations found in PCFS5). The predicted polypeptides lack important functional domains that are seen in the other PCFS orthologs (PCFS2 and PCFS4; **Figure 6A**). These observations raise the possibility that these two genes may not be functional, even in accessions with no clear debilitating changes. Other reports and data support this conclusion. The PCFS1 gene was among those noted in an earlier study as being affected by APA, with a majority of mRNAs encoded by this gene ending well within the protein coding region of the gene (Parker et al., 2021). Such APA products would lack translation termination codons and thus would be substrates for non-stop RNA decay. To confirm that this is the case, different poly(A) site-profiling datasets were analyzed. These data sets include four published ones as well as one independently-generated, hitherto unpublished set of data (see Methods). The results showed that, in every dataset analyzed, a large majority of PCFS1-encoded RNA isoforms end within the protein-coding region of the gene (**Figure 6B**). Similar results were seen in mappings of reads to the PCFS5 gene (**Figure 6C**). These results indicate that most PCFS1- and PCFS5- encoding transcripts are non-stop RNAs. These collective features – the large numbers of missense variants, the occurrence of premature termination codons and frameshift variants, and the prominence of nonstop RNAs encoded by these two genes – strongly suggest that these two *Arabidopsis* genes are not functional, and probable pseudogenes.

Most plants have single genes that encode the Clp1 ortholog, but *Arabidopsis* possesses two such genes, termed CLPS3 and CLPS5 (Hunt et al., 2012). The CLPS3 gene is orthologous to ones that are ubiquitous in plants. This gene is essential (Xing et al., 2008a) and exhibits a very small *π_N_/π_S_* ratio (**Figure 3**). In contrast, the CLPS5 gene seen only in the *Arabidopsis* lineage (Hunt et al., 2012). In the collection of *Arabidopsis* accessions, seven nonsense mutations were found in CLPS5 genes. One nonsense variant (Q97*) was seen in 547 lines (**Table 3**). The expression level of the CLPS5 gene in *Arabidopsis* is very low (**Figure 6D**). Moreover, the *Arabidopsis* CLPS5 is not essential (Xing et al., 2008a). Together, these results suggest that, as with the PCFS1 and PCFS5 genes, CLPS5 is a pseudogene.

#### 3.3.3 PAPS3 – a novel plant poly(A) polymerase borne of paradoxes

Plants possess a conserved set of poly(A) polymerase isoforms, typified by the *Arabidopsis* PAPS1, PAPS2, PAPS3, and PAPS4 proteins (Hunt et al., 2008; Meeks et al., 2009; Hunt et al., 2012; Trost et al., 2014; Kappel et al., 2015; Czesnick and Lenhard, 2016; Zhang et al., 2019). PAPS1, PAPS2, and PAPS4 are all nucleus-localized proteins that play roles in poly(A) tail length control as related to aspects of plant growth and development (Vi et al., 2013; Trost et al., 2014; Kappel et al., 2015; Czesnick and Lenhard, 2016; Zhang et al., 2019; Ramming et al., 2023). These proteins, while related, are functionally-specialized, with specific roles attributed to novel C-terminal domains (Czesnick and Lenhard, 2016). Consistent with this, most of the missense changes in these proteins lie within the respective C-termini (**Figure 4B**; **Supplementary Figures 2B**, **5**); this distribution helps to explain the elevated *π_N_/π_S_* ratios seen with PAPS1 and PAPS2 (**Figure 3**; note that PAPS4 could not be analyzed using the ViVa tool).

**Figure 7:**
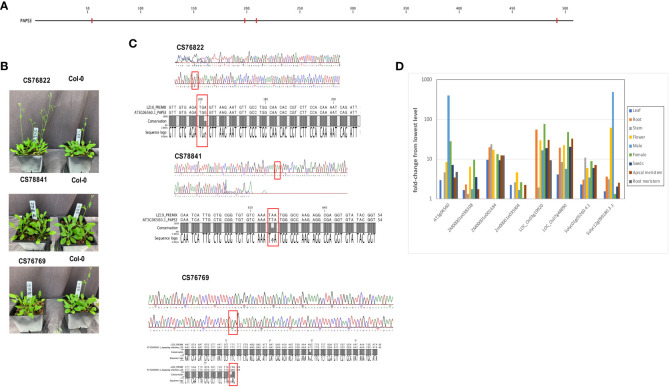
Characterization of ecotypes with nonsense mutations in the PAPS3 gene. **(A)** Locations of pre-mature stop codons seen the *Arabidopsis* 1001 Genomes collection of *Arabidopsis* ecotypes. Nonsense mutations are denoted with red tics. **(B)** Appearances of three ecotypes that harbor nonsense mutations in their respective PAPS3 genes. **(C)** Sanger sequencing traces obtained from DNA isolated from the CS76822, CS78841 and CS76769 ecotypes. Regions from the respective PAPS3 genes that include the stop codons are shown. Pre-mature stop codons are highlighted with red rectangles. The alignments beneath the traces show comparisons of the Col-0 and different ecotype sequences, confirming the alterations that are noted in the *Arabidopsis* 1001 Genomes database. **(D)** Expression characteristics of PAPS3-like genes in different plant species. Normalized expression of the indicated genes in different tissues were determined using data downloaded from the ENDOREPRO database as described in Methods. Normalized values were plotted as shown. Descriptions of the gene designations and the raw data for the plot is provided in **Supplementary File 6**.

In the AtPAPS3 gene, premature stop codons can be found at four different locations in the collection of *Arabidopsis* accessions (**Figure 7A**); these variations are seen (collectively) in 18 different ecotypes (**Table 3**). These stop codons are predicted to severely truncate the encoded proteins and would be null mutations. This was unexpected, as it had been reported that other *Arabidopsis* mutants with PAPS3 null mutations were not viable (Meeks et al., 2009). To explore this, three of these accessions (CS76822, CS76769, CS78841) were grown and characterized. All three accessions had normal growth habits, flowering behaviors, and fertility (**Figure 7B**; **Supplementary Figure 4**). The DNA sequences of the affected sites were subsequently determined after PCR amplification and cloning. The results confirmed the presence of the mutation in a homozygous state (**Figure 7C**). To test the possibility that the PAPS3 gene in these lines had been duplicated, the raw re-sequencing data for these accessions were re-assembled and the assembly searched to identify all contigs that may possess PAPS3-related sequences. This exercise yielded a single contig for each accession (**Supplementary File 5**). These results indicate that there are no additional PAPS3 genes that lack stop codons in these accessions. Several accessions also possess frameshift mutations in the PAPS3 gene - see **Table 4**; **Supplementary File 1**, and **Supplementary Figure 3**. The locations of many of these would dramatically truncate the protein. Given the results seen with the CS76822, CS76769, CS78841 accessions, none of these additional frameshift lines further analyzed.

An earlier bioinformatics analysis reported that the expression of the *Arabidopsis* PAPS3 gene was pollen-specific (Hunt et al., 2008), suggestive of a novel role for this protein in male gametophyte development. Given the features of the PAPS3 gene seen in the collection of *Arabidopsis* accessions, this issue was revisited. For this, PAPS-like genes in a select set of plant species were identified by BLASTP and their expression characteristics compared. The results corroborated the earlier report (Hunt et al., 2008) showing a strong pollen-specific expression of the *Arabidopsis* PAPS3 in pollen (**Figure 7D**; **Supplementary File 6**). One of the two *S. lycopersicum* PAPS3 isoforms (*Solyc12g099180.3.1*) also showed a strong preferential expression in male reproductive tissues as well as in flowers (**Figure 7D**; **Supplementary File 6**). However, the other *S. lycopersicum* PAPS3 isoform, as well as five PAPS3 isoforms present in the maize and rice genomes, did not exhibit strong tissue-specificity in their expression characteristics (**Figure 7D**; **Supplementary File 6**). Therefore, the novel tissue specific expression of the *Arabidopsis* PAPS3 gene is not a universal feature of PAPS3 genes in plants; rather, different genes exhibit different expression properties.

## 4 Discussion

### 4.1 Conservation and variation in subunits of the plant polyadenylation complex – general considerations

For the most part, the patterns of sequence variation that are seen in the *Arabidopsis* genes that encode PAC subunits are consistent with the functioning of these subunits in a fundamental step in gene expression. With the interesting exceptions discussed in the following subsections, the paucity of nonsense and frameshift mutations that would eliminate gene function also supports important roles for these proteins. In some cases, residues seen in most *Arabidopsis* accessions but absent in Col-0 are identical to residues seen in most other plants. This reinforces the point that the Col-0 reference sequence is not necessarily the universal one for *Arabidopsis* or plants in general.

The ratios of non-synonymous to synonymous substitutions (*π_N_/π_S_*) in PAC-associated genes are interesting. As explained in Hamm et al. (Hamm et al., 2019), the *π_N_/π_S_* ratio is a useful proxy for the K_a_/K_s_ metric; thus, low *π_N_/π_S_* ratios may be taken as evidence for purifying selection, and high *π_N_/π_S_* ratios for positive (diversifying) selection. Many PAC-associated genes exhibit high *π_N_/π_S_* ratios. This suggests that many PAC subunits may be under diversifying selection, perhaps evolving in ways to add or alter protein-protein interactions. This is a feature often seen in genes encoding regulatory proteins. Alternative polyadenylation is an important determinant of gene regulation, and the distinctive distribution of PAC-associated genes suggests that a surprising number of these subunits may play roles in alternative polyadenylation.

### 4.2 Multi-purposing is reflected in patterns of variation and sequence diversity

Genes that encode ten PAC subunits possess *π_N_/π_S_* ratios of less than 0.8. These genes encode CPSF160, CPSF100, CPSF73, CLPS3, CFIS2, FIPS5, CFIm68-1, symplekin, PABN1, and PABN2. Interestingly, all but one of the genes that have been reported as essential for plant growth are members of this set. For the one exception, FY, the bulk of divergence resides in the plant-specific C-terminal domain, with the evolutionarily-conserved core of the protein exhibiting a similar paucity of divergence (**Figure 4A**).

A recurring theme in the functioning of many of these relatively invariant PAC subunits is their demonstrated or hypothetical involvement in different RNA processing or metabolic activities apart from their roles in mRNA polyadenylation. For example, CLPS3 is the *Arabidopsis* ortholog of Clp1, a subunit of CFIIm (Xing et al., 2008a; Hunt et al., 2012). Clp1 also plays vital roles in tRNA maturation (Weitzer and Martinez, 2007; Ramirez et al., 2008). Clp1 may act as a more general RNA kinase, as it has been reported to be the kinase responsible for 5’-phosphorylation of siRNAs in mice (Fujinami et al., 2020). CFIS2 is one of two *Arabidopsis* orthologs of CFIm25. CFIS2 (but not CFIS1) also has roles in ribosomal RNA processing (Palm et al., 2019).

CPSF73 is among the most widely-conserved of all subunits of the PAC, being readily identifiable in virtually all eukaryotic genomes. CPSF73 is the enzyme that processes the pre-mRNA prior to polyadenylation. Its activity is tightly regulated, and access to the RNA substrate is controlled through a network of interactions with other PAC subunits. Chief among these subunits is CPSF100, also a protein that shows limited variability in *Arabidopsis* accessions. These two subunits form the core of the endonuclease module of the yeast and mammalian PACs. This module has other functions. For example, in yeast, it also mediates 3’-end processing of snoRNAs (Larochelle et al., 2018). In mammals, it mediates 3’-end formation of histone mRNAs, in conjunction with a dedicated complex that includes the snRNP U7 (Dominski et al., 2005; Kolev and Steitz, 2005). In plants, CPSF73 also plays roles in 3’ end formation of snRNAs (Liu et al., 2016), perhaps analogous to the functioning of the endonuclease module in snoRNA processing in yeast.

Two of the three genes encoding PABN (PABN1 and PABN2) exhibit among the lowest ratios of all PAC subunit-encoding genes. In mammals, PABN helps to control the lengths of poly(A) tracts added to the newly-processed mRNA in the nucleus. PABN also functions in alternative polyadenylation. In *Arabidopsis*, PABN2 has been reported to bind to the C-terminal extension telomerase-reverse transcriptase (TERT), a metallothionein (MTA2), Modifier Of Snc1 (MOS1), a nuclear DNA-binding protein (GP2), Oxidation Related Zinc Finger 2 (OZF2), and a Heat Shock 70 Cognate protein (HSP70-1) (Lee et al., 2012; Dokladal et al., 2015). All three *Arabidopsis* PABN isoforms interact with the *Arabidopsis* Cold Shock Domain 3 (AtCSD3) protein (Kim et al., 2013). The significance of these interactions is not known, but the different interacting partners are not 3’ end processing factors (as far as has been reported). Thus, as is the case with CLPS3, CFIS2, CPSF100, and CPSF73, PABN1 and PABN2 may well have roles apart from those in mRNA polyadenylation.

Based on these considerations, it is tempting to speculate multifunctionality may impose stringent constraints on the abilities of proteins to explore sequence space, such that even modest missense changes may be selected against sufficiently to preclude fixation of variants in populations. This could suggest similar multifunctionality for the other two proteins whose diversity metrics are low. These two proteins, CPSF160 and FIPS5, are scaffolds of sorts. In mammals, CPSF160 coordinates the binding of two other CPSF subunits – CPSF30 and FY – to the polyadenylation signal and serves as a bridge between the PAS-binding module and the so-called cleavage module that consists of CPSF100 and CPSF73. FIP1 (the mammalian and yeast counterpart of FIPS5) recruits poly(A) polymerase to the PAC through interactions with both PAP and CPSF30 (Kumar et al., 2021; Muckenfuss et al., 2022). Analogous interactions have been reported for FIPS5 (Forbes et al., 2006; Hunt et al., 2008), as has a FIPS5-RNA interaction similar to that seen with the mammalian FIP1 ortholog (Forbes et al., 2006). In all three model organisms, the FIP1-CPSF30 interaction involves a conserved zinc finger motif (the C terminal most of the three such motifs in the *Arabidopsis* protein). The *Arabidopsis* FIPS5 protein has a stimulatory effect on the non-specific activity of recombinant PAPS2 (Forbes et al., 2006), and also inhibits a novel endonuclease activity associated with the third zinc finger motif of CPSF30 (Addepalli and Hunt, 2007). Different domains of FIPS5 are associated with interactions with PAP and CPSF30 and with RNA (Forbes et al., 2006). This multiplicity of interactions and activities may impose constraints that limit the sequence diversity seen in the FIPS5 gene in *Arabidopsis* accessions.

### 4.3 Proteins exhibiting high sequence diversity – suggestions of functional specialization

At the other end of the spectrum of sequence diversity are 13 PAC subunits whose *π_N_/π_S_* ratios are greater than 1.5 (**Figure 3**). Three of these (CLPS5, PCFS5, and PAPS3) are also affected by frameshifts and nonsense mutations (**Tables 3**, **4**) and are discussed in following subsections. For the other 10, the patterns of diversity raise intriguing possibilities. These arise because, as indicated by Hughes (Hughes, 1999; Hughes et al., 2000), *π_N_/π_S_* ratios substantially greater than 1 are indicators of positive evolution. Positive evolution is often associated with diversification of protein function, as might be expected for protein isoforms derived from duplicated genes.

Four subunits with *π_N_/π_S_* ratios greater than 1.5 – CFIm68-2, PAPS1, FIPS3, and CFIS1 – are encoded by members of small gene families. For CFIm68-2, FIPS3, and CFIS1, the other members of the gene families (CFIm68-1, FIPS5, and CFIS2) show very low diversity (**Figure 3**). These observations are consistent with the hypothesis that the three genes with high *π_N_/π_S_* ratios encode proteins that possess functions apart, or differently, from their invariant counterparts. PAPS1 is one of three nuclear PAP isoforms; one of these (PAPS4) could not be assessed using the ViVa tool, but the other (PAPS2) showed a moderate degree of diversity, with a *π_N_/π_S_* ratio close to 1. The various PAPS isoforms have been shown to be functionally specialized, with these specialized roles being attributable to the C-termini of the respective proteins. The patterns of diversity seen in the three nuclear PAP isoforms are consistent with this, in that most of the variation seen in *Arabidopsis* accessions is localized to the respective C-terminal domains (**Figure 4B**; **Supplementary Figures 2B**, **5**). These prior demonstrations of specialization amongst *Arabidopsis* nuclear PAP isoforms are consistent with the possibility raised by the high *π_N_/π_S_* ratios seen in the PAPS1 and PAPS2 genes. This in turn lends credence to the proposal that CFIm68-2, FIPS3, and CFIS1 also have distinct (if as yet unknown) roles.

Two of the proteins whose genes exhibit high *π_N_/π_S_* ratios (PCFS2 and PCFS4) encode isoforms of Pcf11. In contrast to the gene pairs represented by CFIS1/CFIS2, FIPS3/FIPS5, and CFIm68-1/CFIm68-2, both *Arabidopsis* Pcf11 isoforms are encoded by genes that exhibit high sequence diversity. This diversity falls outside of the parts of the proteins that are conserved and comprise functional domains (the polII CTD-interacting domain, or CID, and a zinc finger domain; **Figure 6A**). In mammals and yeast, Pcf11 functions in 3’ end formation, transcription termination, and mRNA export (Birse et al., 1998; Grzechnik et al., 2015; Kamieniarz-Gdula et al., 2019). In *Arabidopsis*, PCSF4 has been implicated in transcription termination (de Felippes et al., 2020), and both PCFS2 and PCFS4 are found in nuclear complexes that include the bulk of the polyadenylation complex (Parker et al., 2021). Beyond these reports, little is known about the full scope of functioning of either Pcf11 isoform in plants. Since the CID and zinc finger domains mediate interactions between Pcf11 and the transcription/polyadenylation machineries in mammals and yeast, the paucity of diversity in these domains in PCFS2 and PCFS4 suggest that these two isoforms perform similar, overlapping functions in concert with the plant transcription/polyadenylation machineries. The patterns of diversity in PCFS2 and PCFS4 suggest that these two proteins engage in additional interactions that are more specific for the two isoforms; these specialized interactions might be attributed to the large swaths of each protein that are unique to the respective isoform. Moreover, given the association of high *π_N_/π_S_* ratios with positive selection during evolution, these two sets of specialized functions may be rapidly evolving. Of course, this is at the moment highly speculative. However, it is of interest to note that, in *Populus euphratica*, QTLs that encompass PCFS4 are associated with variation in shoot length (Zhang et al., 2017). Thus, variation in PCFS4 may be causal for an important crop phenotype. This would lend credence to the proposition that PCFS function may be rapidly evolving in plants.

The *Arabidopsis* genome possesses two genes that encode orthologs of symplekin, a scaffold upon which other subcomplexes assemble. One of these genes – ESP4 – has a relatively high *π_N_/π_S_* ratio, while the other – SYM (At1g27595) – has a *π_N_/π_S_* ratio less than 0.8 (**Figure 3**). Neither of these two are, individually, essential for plant growth and development. ESP4 was first identified as a gene mutant of which exhibit increased transcriptional read-through and altered posttranscriptional gene silencing (Herr et al., 2006); these properties are consistent with functioning in mRNA 3’ end formation and transcription termination. SYM mutants have altered responses to sugars (Zheng et al., 2015). The connection between a presumptive role for SYM in mRNA polyadenylation and sugar responses is not clear, and this protein has not been studied in the context of polyadenylation. Interestingly, ESP4 is present in complexes isolated by affinity purification of CPSF100 (Herr et al., 2006), FPA (Parker et al., 2021), CstF77 (Antosz et al., 2017), TFIIS (Antosz et al., 2017), and SPT4 (Antosz et al., 2017); in contrast, SYM is only seen in complexes containing SPT4 (Antosz et al., 2017). While the absence of a protein in a copurification analysis may be due many factors, this difference raises the possibility that the two symplekin orthologs may have somewhat different associations or roles.

Two of the high-diversity PAC subunits are CstF64 and CstF50 (**Figure 3**). In contrast to the subunits discussed in the preceding paragraphs, these proteins are encoded by single genes in *Arabidopsis*. As noted in this report (**Figure 5**) and elsewhere (Hunt, 2020), the plant CstF complex is curiously different from its mammalian counterpart; specifically, whereas CstF is essential in mammals, it is dispensable for viability in *Arabidopsis*. CstF64 and CstF77 play general roles in poly(A) site choice in *Arabidopsis*, but are dispensable for large numbers of poly(A) sites (Zeng et al., 2019). For example, these two proteins promote usage of a proximal poly(A) site associated with the COOLAIR antisense RNA but do not seem to have roles in usage of the poly(A) sites that define the 3’ ends of the “sense” FLC transcripts (Liu et al., 2010). The possibility that CstF64 and CstF50 may be subjected to positive (diversifying) selection raises the possibility natural variation in these proteins may be a source for new or altered regulatory behavior.

### 4.4 PAC-encoding genes that possess premature translation termination codons

Several *Arabidopsis* PAC genes are affected by the occurrence in one or more accessions of premature stop codons and/or frame-shift mutations that severely truncate predicted protein products. This result was unexpected; by way of comparison, none of the 1815 human genes found to be tolerant of biallelic variation impact any of the known subunits of the human PAC (Karczewski et al., 2020). These instances pose questions regarding the structure and functioning of the plant polyadenylation complex. The implications of these results are discussed in the following.

#### 4.4.1 CstF50 is not essential in *Arabidopsis*

The apparent absence of a functional CstF50 gene in one *Arabidopsis* accession (CS77397) that otherwise has a normal growth habit is interesting, since CstF50 is a subunit of a heteromeric complex (CstF) that in mammals is required for mRNA polyadenylation. The mammalian CstF recognizes functional RNA sequences (the DownStream Element, or DSE) 3’ of the cleavage/polyadenylation site. As part of the complex, CstF50 serves to fine-tune the association of the complex with G/U-containing RNAs (that comprise the downstream element) (Yang et al., 2018) and links 3’ end processing with DNA repair (Kleiman and Manley, 1999). In plants, CstF50 is present in nuclear complexes affinity-purified using tagged CstF77 or CstF64 (Antosz et al., 2017), suggestive of a presence in an analogous heteromeric complex. However, it does not seem to interact with the other two CstF subunits in pairwise interaction assays (Yao et al., 2002; Hunt et al., 2008). Beyond these reports, little is known about possible functions of CstF50 in polyadenylation in plants, or the architecture that links CstF50 with CstF77/CstF64-containing nuclear complexes in plants.

These considerations aside, the seeming dispensability of CstF50 in the CS77397 accession aligns with reports indicating that CstF77 and CstF64 are not required for *Arabidopsis* growth and development. Specifically, it has been shown that *Arabidopsis* (Col-0) mutants with null mutations in genes that encode these CstF subunits are viable, if diminished in stature and general growth habit (Zeng et al., 2019). These mutants exhibit a range of phenotypes that may be linked with altered responses to auxin. They also possess a molecular phenotype in which mRNA poly(A) site choice is altered on a genome-wide scale; this phenotype is consistent with the presumed functions of the proteins in polyadenylation. However, the dispensability of these two proteins suggests that CstF may not be needed for the basic functionality of the PAC, namely recognition of the pre-mRNA, endonucleolytic cleavage, and addition of the poly(A) tract.

These considerations notwithstanding, there are some distinctions that may be made. The CstF77 and CstF64 null mutants in the Col-0 background have profound growth phenotypes. CS77397, in contrast, has a growth habit that is as unremarkable as most other *Arabidopsis* accessions, and shows no hints of having strongly-altered auxin responses. Moreover, if one grants a cause-and-effect relationship between the growth phenotypes, altered auxin responses, and global changes in poly(A) site choice in the CstF77 and CstF64 mutants, it stands to reason that global poly(A) site choice is probably not affected by the absence of CstF50 in the CS77397 accession. This in turn suggests a modest role for CstF50 in the functioning of the PAC. Other eukaryotic lineages lack CstF50 orthologs; these lineages include yeast, in which two other orthologs of CstF subunits (Rna14 and Rna15) function as part of a complex (CF1A) that lacks a CstF50 ortholog. It may be that the plant PAC may be more akin to the yeast than the mammalian complex, and the plant CstF50 may be an accessory rather than a core PAC subunit.

Clearly, the functioning of CstF50 in polyadenylation is largely undefined, with much remaining to be learned. Whatever its role(s), the variability beyond the singular premature termination codon in CS77397 raises some interesting possibilities. In particular, the high *π_N_/π_S_* ratio seen in the CstF50 gene suggests that this protein may be subject to diversifying evolutionary change. Such a possibility is consistent with a role as an accessory protein in the complex, one whose activity (or even presence) may vary in the plant and over evolutionary time.

The existence of an *Arabidopsis* accession that has a nonfunctional CstF50 gene raises questions as to how CstF50 might persist over evolutionary time in the plant lineage. The durability of the plant CstF50, even after many millions of years of evolution, strongly suggests that the protein has important functions that are targets of natural selection. This possibility is not consistent with the dispensability of the protein in the CS77397 accession. It is difficult to resolve this paradox at the moment. However, this curious result magnifies the possibility that the plant CstF50 has unexpected roles, either in mRNA polyadenylation or perhaps other aspects of plant growth and development.

#### 4.4.2 PCFS1/PCFS5 (Pcf11) and CLPS5 (Clp1) are pseudogenes

*Arabidopsis* possesses three genes encoding PAC subunits that are not seen in other plants; these are genes that encode novel orthologs of Pcf11 and Clp1, subunits of CFIIm. Two of these genes, termed PCFS1 and PCFS5, encode novel Pcf11-related proteins. In a survey of 11 well-characterized plant genomes, these two genes were only seen *Arabidopsis thaliana*, *Arabidopsis lyrata*, and perhaps *Populus trichocarpa* (Hunt et al., 2012). The genes encoding PCFS1 and PCSF5 are similar in gene structure. They lack introns and a majority of RNAs specified by the *Arabidopsis* genes terminate at distinct poly(A) sites situated well within the respective protein-coding regions (**Figure 6**). As such, these RNAs would likely be substrates for nonstop RNA degradation. The PCFS1 and PCFS5 genes are also impacted by frameshift and nonsense mutations in the collection of *Arabidopsis* accessions. The polypeptides encoded by the full-length PCFS1 and PCFS5 mRNAs are truncated when compared with other *Arabidopsis* Pcf11 orthologs (PCFS2 and PCFS4 mentioned in the preceding) and lack the CID domains seen in the other Pcf11 orthologs. Collectively, these data raise the possibility that the genes that encode PCFS1 and PCFS5 are likely to be pseudogenes.

Like PCFS1 and PCFS5, the novel Clp1-related isoform CLPS5 seems to be specific for the *Arabidopsis* lineage, and is not found in other plant genomes (Hunt et al., 2012). In contrast to CLPS3, *Arabidopsis* mutants with T-DNA insertions that would disrupt the CLPS5 gene are viable, indicating that this protein is dispensable for growth and development (Xing et al., 2008a). 590 *Arabidopsis* accessions were found to possess CLPS5 genes with premature termination codons, most (or all) of which would dramatically truncate translated polypeptides (**Table 3**; **Figure 6**). In addition, surveys of gene expression indicate that the CLPS5 gene is expressed at very low levels, if at all (Hunt et al., 2008). Taken together, these observations suggest that the *Arabidopsis* CLPS5 gene is a lineage-specific duplicate that lacks function and is likely a pseudogene.

#### 4.4.3 PAPS3 - an enigma

Of the polyadenylation-associated genes in the *Arabidopsis* genome, PAPS3 is perhaps the most perplexing. PAPS3-like proteins, enzymes that lack C-terminal domains that are associated with nuclear polyadenylation and specialized functions of nuclear PAPs in *Arabidopsis*, are widespread in the plant lineage (Hunt et al., 2012). However, the variation seen the *Arabidopsis* PAPS3 gene is extensive, with a high *π_N_/π_S_* ratio and premature termination codons in many accessions (**Figure 3**; **Table 3**). These features are similar to those seen in the PCFS1, PCFS5, and CLPS5 genes, and thus raise the possibility that PAPS3 genes may be non-functional, and perhaps pseudogenes. These observations are cause to re-visit other aspects of PAPS3 genes in plants. For example, in contrast to PCFS1, PCFS5, and CLPS5, all of which are seen only in *Arabidopsis*, PAPS3-like genes are found widely in angiosperms (Hunt et al., 2012). However, their occurrence is not universal, as some species (for example, *Glycine max*) lack identifiable PAPS3-like genes (Hunt et al., 2012). Therefore, PAPS3-like proteins are likely not an essential part of the plant proteomic toolkit. The observation that the expression of the *Arabidopsis* PAPS3 gene is strongly pollen-specific suggested a role for the protein in some aspect of male gametophyte development (Hunt et al., 2012). However, male-specific expression is not a general feature of plant PAPS3-like genes (**Figure 7D**).

As is the case with CstF50, it is challenging to reconcile the evolutionary conservation of PAPS3 genes in plants with the dispensability of the protein in *Arabidopsis* (documented in this report) and its seeming absence in other plants. If one assumes that PAPS3 was present in the common ancestor of higher plants, its absence in species such as *Glycine max* supports the contention that, absent selectable roles, this gene is subject to evolutionary forces (random mutant, chiefly) that over the course of time would eliminate the gene. Given that the *Arabidopsis* PAPS3 gene is not essential (this study) and can be inactivated without obvious phenotypic impacts, it is reasonable to expect that this gene should not persist, but rather should be lost in higher plants. However, this is not the case. Along with the distinct and different PAPS3 expression patterns noted here (**Figure 7D**), these results raise the possibility that PAPS3 orthologs may have evolved lineage-specific functions that are both dispensable (at least in some cases, as is seen in *Arabidopsis*) and subject to natural selection (so as to preserve the genes over evolutionary time scales).

## 5 Summary

We have compiled and studied the range of variation in *Arabidopsis thaliana* that affects the different subunits of the polyadenylation complex. The results suggest that a sizable number of PAC subunits exhibit variation that is suggestive of a degree of diversifying selection, and may indicate expanded roles for different subunits in the regulation of alternative polyadenylation. At least three genes, all *Arabidopsis*-specific, are likely to non-functional, based on both the widespread occurrence of disruptive (e.g., nonsense) mutations and gene expression patterns that are consistent with a lack of function. Most interestingly, two genes (CstF50 and PAPS3) that are widely-conserved in plants are affected in some accessions by disruptive mutations. The seeming dispensability of these genes is difficult to reconcile by their broad evolutionary conservation, and poses new questions regarding the composition and functioning of the plant polyadenylation complex.

## Data availability statement

The high throughput sequencing data generated in the course of this research may be found under Bioproject PRJNA1023006. Accessions for the publicly available datasets analyzed in this work are provided in **Supplementary File 8**. The original contributions presented in the study are included in the article and **Supplementary Material**; further inquiries can be directed to the corresponding authors.

## Author contributions

LZ: Data curation, Formal analysis, Investigation, Methodology, Validation, Writing – original draft, Writing – review & editing. KL: Data curation, Investigation, Methodology, Writing – review & editing. AH: Conceptualization, Data curation, Formal analysis, Funding acquisition, Investigation, Methodology, Project administration, Resources, Supervision, Validation, Visualization, Writing – original draft, Writing – review & editing.
