# Supplementary material for: Natural variation in the plant polyadenylation complex
Source: Front Plant Sci. 2024 Jan 22;14:1303398. doi: 10.3389/fpls.2023.1303398 (PMC10839035; doi:10.3389/fpls.2023.1303398)
Supplement: Supplementary file 1 [file DataSheet_1.pdf]

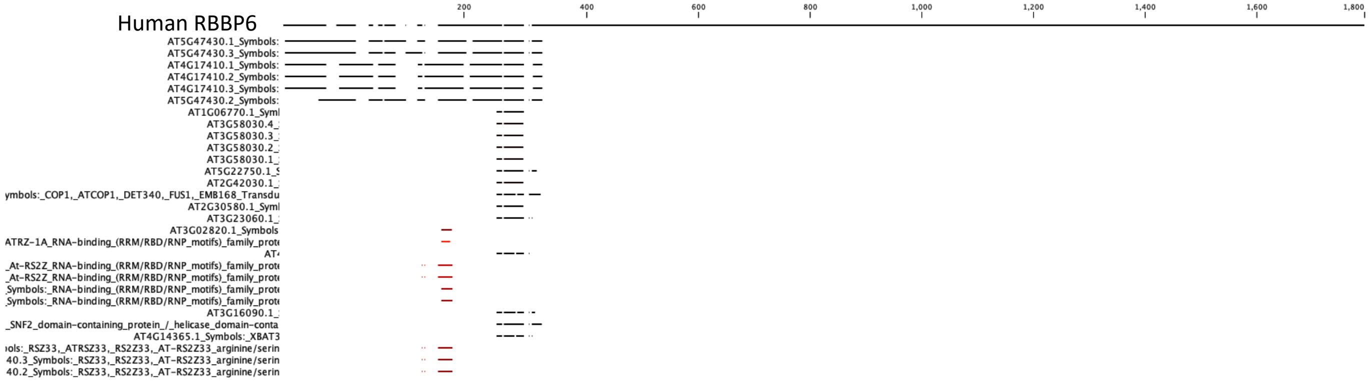

Supplementary FIGURE 1 RBBP6 similar genes found in Arabidopsis. Human RBBP6 amino acid as query to blast Col-0 protein database. The first line shows the human RBBP6 amino acid, the similar genes found in Col-0 were showed below, the text in the left represent the gene name found in Col-0, and the black and red truncate lines in each gene represent the overlap region with Human RBBP6.

A

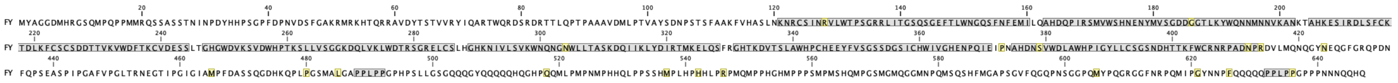

B

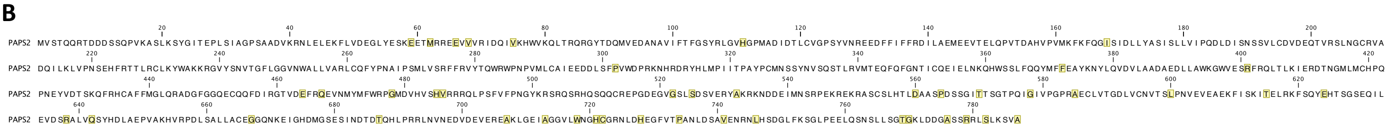

Supplemental FIGURE 2 FY and PAPS3 amino acid sequence and missense mutation position in each gene. **(A)** WD domain and PPLPP motifs are highlighted with grey boxes, positions affected by point mutations in different Arabidopsis ecotypes with yellow shading. **(B)** Amino acid sequence of the Arabidopsis PAPS3 protein. The positions affected by point mutations in different Arabidopsis ecotypes with yellow shading.

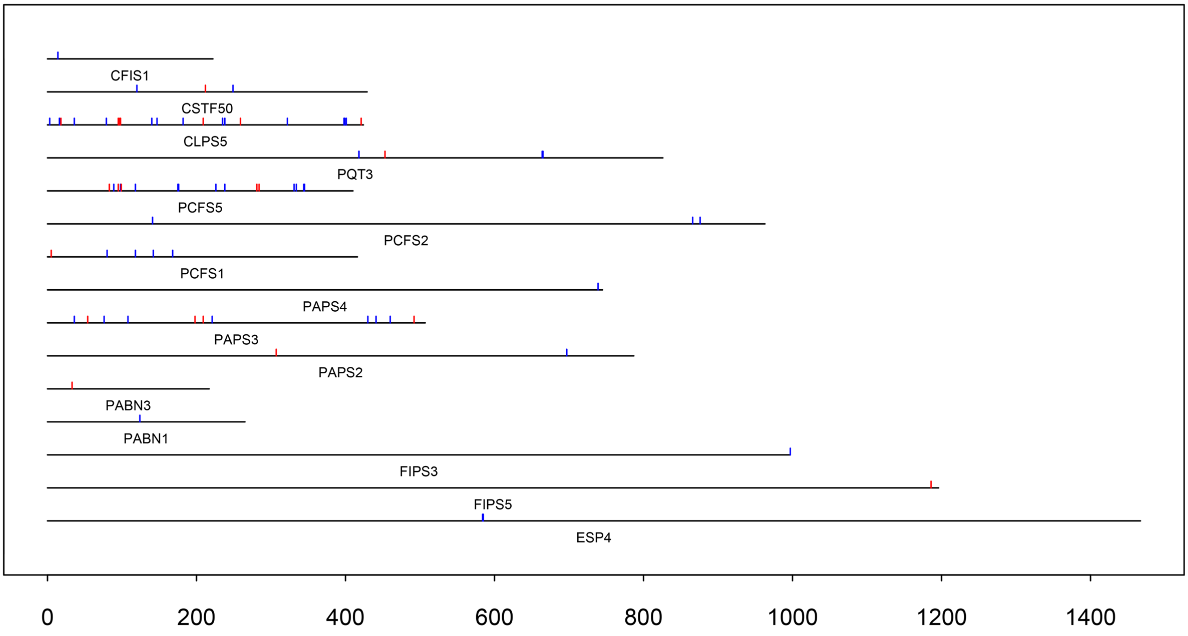

Supplemental FIGURE 3 Nonsense and frameshift in each gene. Red color and blue color denotes pre-mature stop codon or frameshift (insertion or deletion) found in the gene, respectively. The x axis shows the gene length.

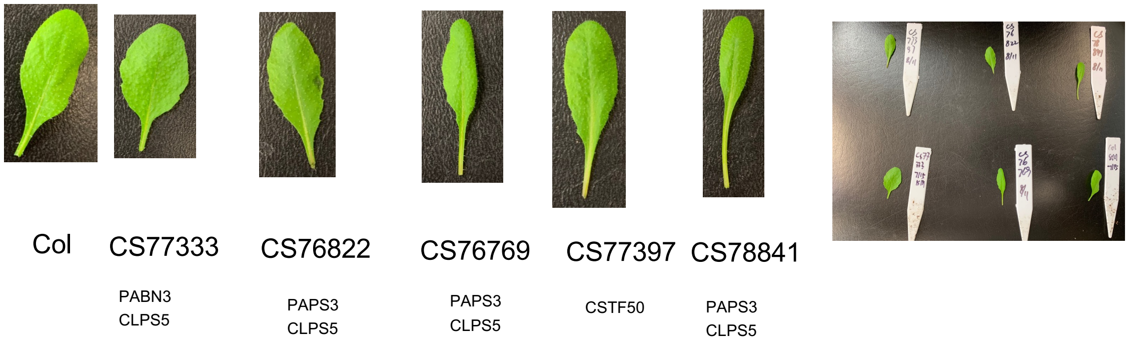

Supplemental FIGURE 4 Five different ecotypes contain different pre-mature stop codon and their leaves phenotype compare with Columbia-0. The name of ecotypes listed below the picture, and the pre-mature stop codon gained gene listed in the bottom. Four of them contains two different PAC genes.

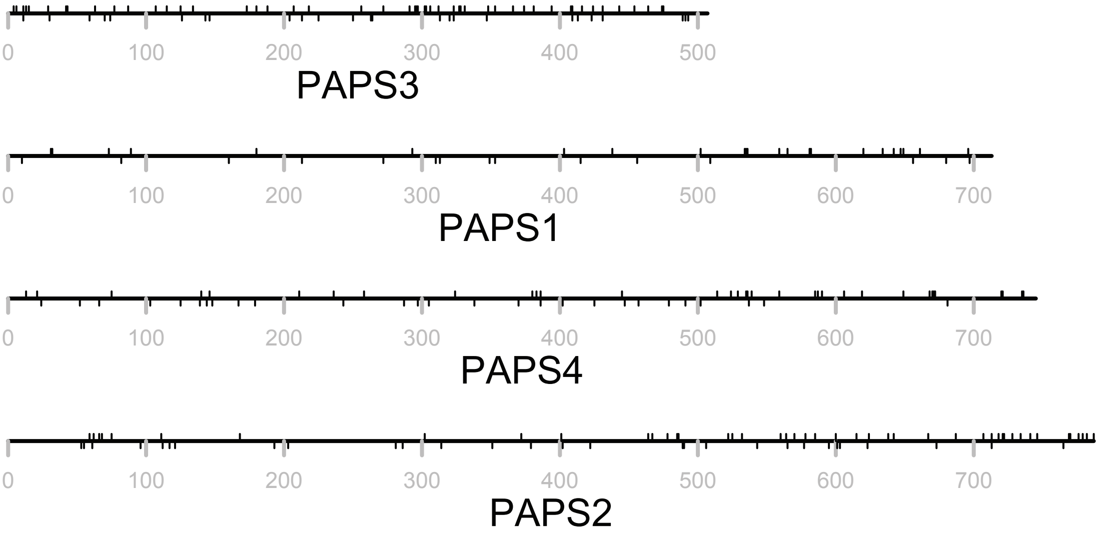

Supplemental FIGURE 5 Highlight the missense and silent point mutation in each PAC. The black lines above the gene length are missense mutations, and below are silent mutations. Grey line and text represent the gene length scale
