# Supplementary material for: Natural variation in the plant polyadenylation complex
Source: Front Plant Sci. 2024 Jan 22;14:1303398. doi: 10.3389/fpls.2023.1303398 (PMC10839035; doi:10.3389/fpls.2023.1303398)
Supplement: Supplementary file 5 [file Table_4.docx]

Supplemental File 4 – Results showing the reassembly and BLAST analysis for the identification of possible CstF50-related genes in Arabidopsis Strain 9812.

A. Depiction of the BLAST results, provided by CLC Genomics Workbench.

B. BLAST results table.

| Query sequence | Hit | E-value | Score | Bit score |
| --- | --- | --- | --- | --- |
| CTF50_ARATH | sample_9812_contig_6174 | 2E-135 | 536 | 211.075 |
| CTF50_ARATH | sample_9812_contig_6174 | 2E-135 | 435 | 172.17 |
| CTF50_ARATH | sample_9812_contig_6174 | 2E-135 | 254 | 102.449 |
| CTF50_ARATH | sample_9812_contig_6174 | 2E-135 | 118 | 50.0618 |
| CTF50_ARATH | sample_9812_contig_6174 | 2E-135 | 73 | 32.7278 |
| CTF50_ARATH | sample_9812_contig_6174 | 1.92E-33 | 159 | 65.855 |
| CTF50_ARATH | sample_9812_contig_6174 | 1.92E-33 | 117 | 49.6766 |
| CTF50_ARATH | sample_9812_contig_6174 | 1.92E-33 | 104 | 44.669 |
| CTF50_ARATH | sample_9812_contig_6174 | 1.92E-33 | 100 | 43.1282 |
| CTF50_ARATH | sample_9812_contig_6174 | 4.06E-15 | 194 | 79.337 |
| CTF50_ARATH | sample_9812_contig_7913 | 5.31E-10 | 153 | 63.5438 |
| CTF50_ARATH | sample_9812_contig_7913 | 2.9E-07 | 130 | 54.6842 |
| CTF50_ARATH | sample_9812_contig_35831 | 7.17E-07 | 119 | 50.447 |
| CTF50_ARATH | sample_9812_contig_35831 | 1.87E-05 | 108 | 46.2098 |
| CTF50_ARATH | sample_9812_contig_35831 | 5.36E-05 | 105 | 45.0542 |
| CTF50_ARATH | sample_9812_contig_461 | 7.38E-06 | 118 | 50.0618 |
| CTF50_ARATH | sample_9812_contig_461 | 0.336977 | 79 | 35.039 |
| CTF50_ARATH | sample_9812_contig_461 | 1.29999 | 74 | 33.113 |

C. Blast results – copied exactly from the BLAST output.

ALIGNMENTS

>sample_9812_contig_6174 Average coverage: 16.02

Length=8120

Score = 211.1 bits (536), Expect = 2E-135

Identities = 107/135 (79%), Positives = 107/135 (79%), Gaps = 28/135 (21%)

Frame = +1

Query 323 RFVLSSGKDSTVKLWEIGSGRMVKEYLGAKRVKLRSQ----------------------- 359

RFVLSSGKDSTVKLWEIGSGRMVKEYLGAKRVKLRSQ

Sbjct 3118 RFVLSSGKDSTVKLWEIGSGRMVKEYLGAKRVKLRSQVTRFLNLLRQKYSLRRLILTGLH 3297

Query 360 -----AIFNDTEEFVISIDEASNEVVTWDARTADKVAKWPSNHNGAPRWIEHSPVESVFV 413

AIFNDTEEFVISIDEASNEVVTWDARTADKVAKWPSNHNGAPRWIEHSPVESVFV

Sbjct 3298 CCVLQAIFNDTEEFVISIDEASNEVVTWDARTADKVAKWPSNHNGAPRWIEHSPVESVFV 3477

Query 415 TCGIDRSIRFWKESV 429

TCGIDRSIRFWKESV

Sbjct 3478 TCGIDRSIRFWKESV 3522

Score = 172.2 bits (435), Expect = 2E-135

Identities = 87/121 (71%), Positives = 87/121 (72%), Gaps = 33/121 (27%)

Frame = +2

Query 236 LAGTDHPIPHLYDVNTYQCFLPSNFPDSGVSGAINQVR---------------------- 273

AGTDHPIPHLYDVNTYQCFLPSNFPDSGVSGAINQVR

Sbjct 2663 FAGTDHPIPHLYDVNTYQCFLPSNFPDSGVSGAINQVRHTLSDKC*LCVL*TSLKMIKDS 2842

Query 274 -----------YSSTGSIYITASKDGAIRLFDGVSAKCVRSIGNAHGKSEVTSAVFTKDQ 321

YSSTGSIYITASKDGAIRLFDGVSAKCVRSIGNAHGKSEVTSAVFTKDQ

Sbjct 2843 WL*DSWFF*VRYSSTGSIYITASKDGAIRLFDGVSAKCVRSIGNAHGKSEVTSAVFTKDQ 3022

Query 323 R 323

R

Sbjct 3023 R 3025

Score = 102.4 bits (254), Expect = 2E-135

Identities = 60/107 (56%), Positives = 66/107 (62%), Gaps = 36/107 (34%)

Frame = +2

Query 136 MFF--ATGGADTSIKL----FEVPKVKQMISGDTQARPLIRTFYDHAE------------ 177

+FF A+ D+S+ + FEVPKVKQMISGDTQARPLIRTFYDHAE

Sbjct 1952 LFFLQASSRVDSSVDIPCCFFEVPKVKQMISGDTQARPLIRTFYDHAEVSVSFFKIFAVL 2131

Query 178 -----------------PINDLDFHPRSTILISSAKDNCIKF-FDFS 205

PINDLDFHPRSTILISSAKDNCIK+ F FS

Sbjct 2132 ILCIS*TGS*ISDNHLQPINDLDFHPRSTILISSAKDNCIKYIFTFS 2272

Score = 50.1 bits (118), Expect = 2E-135

Identities = 21/25 (84%), Positives = 23/25 (92%), Gaps = 0/25 (0%)

Frame = +3

Query 214 FKVFQDTHNVRSISFHPSGEFLLAG 238

++ QDTHNVRSISFHPSGEFLLAG

Sbjct 2481 LQILQDTHNVRSISFHPSGEFLLAG 2555

Score = 32.7 bits (73), Expect = 2E-135

Identities = 16/18 (88%), Positives = 17/18 (94%), Gaps = 0/18 (0%)

Frame = +1

Query 201 KFFDFSKTTAKRAFKVFQ 218

+FFDFSKTTAK AFKVFQ

Sbjct 2353 RFFDFSKTTAK*AFKVFQ 2406

Score = 65.9 bits (159), Expect = 2E-33

Identities = 30/35 (85%), Positives = 31/35 (89%), Gaps = 0/35 (0%)

Frame = +3

Query 124 SVVRCARFSPDGMFFATGGADTSIKLFEVPKVKQM 158

SVVRCARFSPDGMFFATGGADTSIKLFEVP +

Sbjct 1836 SVVRCARFSPDGMFFATGGADTSIKLFEVPNFCHI 1940

Score = 49.7 bits (117), Expect = 2E-33

Identities = 24/27 (88%), Positives = 25/27 (93%), Gaps = 0/27 (0%)

Frame = +1

Query 97 IDFSVNHAKGSSKTIPKHESKTLSEHK 123

+ SVNHAKGSSKTIPKHESKTLSEHK

Sbjct 1639 VRTSVNHAKGSSKTIPKHESKTLSEHK 1719

Score = 44.7 bits (104), Expect = 2E-33

Identities = 20/20 (100%), Positives = 20/20 (100%), Gaps = 0/20 (0%)

Frame = +1

Query 44 TMTPLNIEVPPNRLLELVAK 63

TMTPLNIEVPPNRLLELVAK

Sbjct 1306 TMTPLNIEVPPNRLLELVAK 1365

Score = 43.1 bits (100), Expect = 2E-33

Identities = 36/37 (97%), Positives = 37/37 (100%), Gaps = 0/37 (0%)

Frame = +2

Query 63 KGLAAENNGTLRXXXXXXXXXXXXXXITTPRTASIDF 99

+GLAAENNGTLRGVSSSVLLPSSYGSITTPRTASIDF

Sbjct 1445 QGLAAENNGTLRGVSSSVLLPSSYGSITTPRTASIDF 1555

Score = 79.3 bits (194), Expect = 4E-15

Identities = 38/49 (77%), Positives = 41/49 (84%), Gaps = 0/49 (0%)

Frame = +2

Query 1 MGNSGDLEQALQDGNIFRQLNALIVAHLRHHNLSQVASAVASATMTPLN 49

MGNSGDLEQALQDGNIFRQLNALIVAHLRHHNLSQV S++ + N

Sbjct 962 MGNSGDLEQALQDGNIFRQLNALIVAHLRHHNLSQVCFCYRSSSHSDSN 1108

>sample_9812_contig_7913 Average coverage: 17.65

Length=14861

Score = 63.5 bits (153), Expect = 5E-10

Identities = 73/323 (22%), Positives = 133/323 (41%), Gaps = 28/323 (9%)

Frame = -1

Query 111 IPKHESKTLSEHKSVVRCARFSP-DGMFFATGGADTSIKLFEVPKVKQMISGDTQARPLI 169

IPK T S H V RF P G + G D +K+++V + +

Sbjct 9950 IPKRLVHTWSGHTKGVSAIRFFPKQGHLLLSAGMDCKVKIWDV----------YNSGKCM 9801

Query 170 RTFYDHAEPINDLDFHPRSTILISSAKDNCIKFFDFSKTTAKRAFKVFQDTHNVRSISFH 229

RT+ HA+ + D+ F + +++ D IK++D T + F + +

Sbjct 9800 RTYMGHAKAVRDICFSNDGSKFLTAGYDKNIKYWD---TETGQVISTFSTGKIPYVVKLN 9630

Query 230 PSGE---FLLAGTDHPIPHLYDVNTYQCFLPSNFPDSGVSGAINQVRYSSTGSIYITASK 286

P + LLAG +D+NT + + D + GA+N + + ++T+S

Sbjct 9629 PDDDKQNILLAGMSDKKIVQWDINTGEV---TQEYDQHL-GAVNTITFVDNNRRFVTSSD 9462

Query 287 DGAIRLFDGVSAKCVRSIGNAHGKSEVTSAVFTKDQRFVLSSGKDSTVKLWEIGSGRMV- 345

D ++R+++ ++ I H S + +V + ++ + D+ + ++ +

Sbjct 9461 DKSLRVWEFGIPVVIKYISEPHMHSMPSISVHP-NGNWLAAQSLDNQILIYSTRERFQLN 9285

Query 346 --KEYLGAKRVKLRSQAIFNDTEEFVISIDEASNEVVTWDARTADKVAKWPSNHNGAPRW 402

K + G Q F+ FV+S D + WD ++ KV + HNG

Sbjct 9284 KKKRFAGHIVAGYACQVNFSPDGRFVMSGD-GEGKCWFWDWKSC-KVFRTLKCHNGVCIG 9111

Query 404 IEHSPVE-SVFVTCGIDRSIRFW 425

E P+E S TCG D I++W

Sbjct 9110 AEWHPLEQSKVATCGWDGLIKYW 9042

Score = 54.7 bits (130), Expect = 3E-07

Identities = 45/184 (24%), Positives = 86/184 (47%), Gaps = 14/184 (8%)

Frame = -1

Query 250 NTYQCFLPSNFPD--SGVSGAINQVRY-SSTGSIYITASKDGAIRLFDGV-SAKCVRSIG 305

N C++P SG + ++ +R+ G + ++A D ++++D S KC+R+

Sbjct 9968 NNDHCYIPKRLVHTWSGHTKGVSAIRFFPKQGHLLLSAGMDCKVKIWDVYNSGKCMRTY- 9792

Query 306 NAHGKSEVTSAVFTKDQRFVLSSGKDSTVKLWEIGSGRMVKEYLGAK---RVKLRSQAIF 362

H K+ V F+ D L++G D +K W+ +G+++ + K VKL

Sbjct 9791 MGHAKA-VRDICFSNDGSKFLTAGYDKNIKYWDTETGQVISTFSTGKIPYVVKLNP---- 9630

Query 363 NDTEEFVISIDEASNEVVTWDARTADKVAKWPSNHNGAPRWIEHSPVESVFVTCGIDRSI 422

+D ++ ++ + ++V WD T + ++ H GA I FVT D+S+

Sbjct 9626 DDDKQNILLAGMSDKKIVQWDINTGEVTQEY-DQHLGAVNTITFVDNNRRFVTSSDDKSL 9453

Query 423 RFWK 426

R W+

Sbjct 9449 RVWE 9438

>sample_9812_contig_35831 Average coverage: 16.08

Length=507

Score = 50.4 bits (119), Expect = 7E-07

Identities = 30/125 (24%), Positives = 58/125 (46%), Gaps = 16/125 (13%)

Frame = +1

Query 117 KTLSEHKSVVRCARFSPDGMFFATGGADTSIKLFEVPKVKQMISGDTQARPLIRTFYDHA 176

KTL H + C F+P +G D ++++++V K ++ H+

Sbjct 130 KTLIGHTNYAFCVNFNPQSNMIVSGSFDETVRIWDVTTGK-----------CLKVLPAHS 276

Query 177 EPINDLDFHPRSTILISSAKDNCIKFFDFSKTTAKRAFKVFQDTHN--VRSISFHPSGEF 234

+P+ +DF+ ++++SS+ D + +D + K D N V + F P+G+F

Sbjct 277 DPVTAVDFNRDGSLIVSSSYDGLCRIWD---SGTGHCVKTLIDDENPPVSFVRFSPNGKF 447

Query 235 LLAGT 239

+L GT

Sbjct 448 ILVGT 462

Score = 46.2 bits (108), Expect = 2E-05

Identities = 28/129 (21%), Positives = 64/129 (50%), Gaps = 6/129 (5%)

Frame = +1

Query 221 HNVRSISFHPSGEFLLAGTDHPIPHLYDVNTYQCFLPSNFPDSGVSGAINQVRYSSTGSI 280

+ + ++F F+++ +D L+DV T + + + + +N ++ ++

Sbjct 25 NGISDVAFSSDARFIVSASDDKTLKLWDVETGS-LIKTLIGHTNYAFCVN---FNPQSNM 192

Query 281 YITASKDGAIRLFDGVSAKCVRSIGNAHGKSEVTSAVFTKDQRFVLSSGKDSTVKLWEIG 340

++ S D +R++D + KC++ + AH VT+ F +D ++SS D ++W+ G

Sbjct 193 IVSGSFDETVRIWDVTTGKCLKVLP-AH-SDPVTAVDFNRDGSLIVSSSYDGLCRIWDSG 366

Query 341 SGRMVKEYL 349

+G VK +

Sbjct 367 TGHCVKTLI 393

Score = 45.1 bits (105), Expect = 5E-05

Identities = 33/123 (26%), Positives = 57/123 (46%), Gaps = 11/123 (9%)

Frame = +1

Query 305 GNAHGKSEVTSAVFTKDQRFVLSSGKDSTVKLWEIGSGRMVKEYLGAKRVKLRSQAIFND 364

G+ +G S+V F+ D RF++S+ D T+KLW++ +G ++K +G FN

Sbjct 16 GHENGISDVA---FSSDARFIVSASDDKTLKLWDVETGSLIKTLIGHTNYAFCVN--FNP 180

Query 365 TEEFVI--SIDEASNEVVTWDARTADKVAKWPSNHNGAPRWIEHSPVESVFVTCGIDRSI 422

++ S DE V WD T + P+ H+ ++ + S+ V+ D

Sbjct 181 QSNMIVSGSFDET---VRIWDVTTGKCLKVLPA-HSDPVTAVDFNRDGSLIVSSSYDGLC 348

Query 423 RFW 425

R W

Sbjct 349 RIW 357

>sample_9812_contig_461 Average coverage: 17.49

Length=16768

Score = 50.1 bits (118), Expect = 7E-06

Identities = 26/85 (30%), Positives = 43/85 (51%), Gaps = 11/85 (13%)

Frame = -1

Query 117 KTLSEHKSVVRCARFSPDGMFFATGGADTSIKLFEVPKVKQMISGDTQARPLIRTFYDHA 176

+TL+ H+S F P G FFA+G DT++K++++ K + I T+ H

Sbjct 13741 RTLTGHRSNCVSVNFHPFGEFFASGSLDTNLKIWDIRK-----------KGCIHTYKGHT 13595

Query 177 EPINDLDFHPRSTILISSAKDNCIK 201

+N L F P ++S +DN +K

Sbjct 13594 RGVNVLRFTPDGRWIVSGGEDNVVK 13520

Score = 35.0 bits (79), Expect = 3E-01

Identities = 21/90 (23%), Positives = 36/90 (40%), Gaps = 7/90 (8%)

Frame = -1

Query 168 LIRTFYDHAEPINDLDFHPRSTILISSAKDNCIKFFDFSKTTAKRAFKVFQDTHNVRSIS 227

++RT H ++FHP S + D +K +D K +K T V +

Sbjct 13747 VVRTLTGHRSNCVSVNFHPFGEFFASGSLDTNLKIWDIRKKGCIHTYK--GHTRGVNVLR 13574

Query 228 FHPSGEFLLAGTDHPIPHLYDVNTYQCFLP 257

F P G ++++G + + V C P

Sbjct 13573 FTPDGRWIVSGGEDNV-----VKVCSCLFP 13499

Score = 33.1 bits (74), Expect = 1E00

Identities = 16/71 (22%), Positives = 34/71 (48%), Gaps = 4/71 (6%)

Frame = -1

Query 222 NVRSISFHPSGEFLLAGTDHPIPHLYDVNTYQCFLPSNFPDSGVSGAINQVRYSSTGSIY 281

N S++FHP GEF +G+ ++D+ C G + +N +R++ G

Sbjct 13717 NCVSVNFHPFGEFFASGSLDTNLKIWDIRKKGCI----HTYKGHTRGVNVLRFTPDGRWI 13550

Query 282 ITASKDGAIRL 292

++ +D +++

Sbjct 13549 VSGGEDNVVKV 13517
