# Supplementary material for: Natural variation in the plant polyadenylation complex
Source: Front Plant Sci. 2024 Jan 22;14:1303398. doi: 10.3389/fpls.2023.1303398 (PMC10839035; doi:10.3389/fpls.2023.1303398)
Supplement: Supplementary file 6 [file Table_5.docx]

Supplemental File 5. Results showing the reassembly and BLAST analysis for the identification of possible PAPS3-related genes in Arabidopsis strains CS76822, CS76769, and CS78841.

For each set of results, the upper panel depicts the output in graphical format, showing the alignment of the Col-0 reference with different contigs derived from the re-assembly. The lower panel displays a table of the results, showing the “strengths” of each alignment.

A. Depiction of the results for reassembly of strain CS76822. Different sections are as described in Supplemental Fig. 4.

| Query sequence | Hit | E-value |
| --- | --- | --- |
| At_PAPS3 | SRR1945601_contig_5726 | 3.8E-141 |
| At_PAPS3 | SRR1945601_contig_5726 | 3.8E-141 |
| At_PAPS3 | SRR1945601_contig_5726 | 3.8E-141 |
| At_PAPS3 | SRR1945601_contig_5726 | 3.8E-141 |
| At_PAPS3 | SRR1945601_contig_5726 | 3.8E-141 |
| At_PAPS3 | SRR1945601_contig_5726 | 3.8E-141 |
| At_PAPS3 | SRR1945601_contig_5726 | 1E-105 |
| At_PAPS3 | SRR1945601_contig_5726 | 1E-105 |
| At_PAPS3 | SRR1945601_contig_2220 | 2.82E-27 |
| At_PAPS3 | SRR1945601_contig_2220 | 1.33E-10 |
| At_PAPS3 | SRR1945601_contig_2220 | 2.11642 |
| At_PAPS3 | SRR1945601_contig_2769 | 9.43E-27 |
| At_PAPS3 | SRR1945601_contig_2769 | 9.43E-27 |
| At_PAPS3 | SRR1945601_contig_2769 | 3.97E-13 |
| At_PAPS3 | SRR1945601_contig_2769 | 3.60448 |
| At_PAPS3 | SRR1945601_contig_542 | 7.15E-25 |
| At_PAPS3 | SRR1945601_contig_542 | 1.24E-12 |
| At_PAPS3 | SRR1945601_contig_542 | 1.24E-12 |
| At_PAPS3 | SRR1945601_contig_542 | 0.000581 |
| At_PAPS3 | SRR1945601_contig_555 | 3.7E-20 |
| At_PAPS3 | SRR1945601_contig_555 | 3.7E-20 |
| At_PAPS3 | SRR1945601_contig_555 | 3.7E-20 |
| At_PAPS3 | SRR1945601_contig_555 | 7.83E-10 |
| At_PAPS3 | SRR1945601_contig_555 | 7.83E-10 |

B. Depiction of the results for reassembly of strain CS78841. Different sections are as described in Supplemental Fig. 4.

| Query sequence | Hit | E-value |
| --- | --- | --- |
| At_PAPS3 | SRR1946188_contig_4290 | 6.7E-143 |
| At_PAPS3 | SRR1946188_contig_4290 | 6.7E-143 |
| At_PAPS3 | SRR1946188_contig_4290 | 6.7E-143 |
| At_PAPS3 | SRR1946188_contig_4290 | 6.7E-143 |
| At_PAPS3 | SRR1946188_contig_4290 | 6.7E-143 |
| At_PAPS3 | SRR1946188_contig_4290 | 6.7E-143 |
| At_PAPS3 | SRR1946188_contig_4290 | 1E-105 |
| At_PAPS3 | SRR1946188_contig_4290 | 1E-105 |
| At_PAPS3 | SRR1946188_contig_11464 | 2.73E-27 |
| At_PAPS3 | SRR1946188_contig_11464 | 1.17E-10 |
| At_PAPS3 | SRR1946188_contig_11464 | 2.07956 |
| At_PAPS3 | SRR1946188_contig_1459 | 9.33E-27 |
| At_PAPS3 | SRR1946188_contig_1459 | 9.33E-27 |
| At_PAPS3 | SRR1946188_contig_1459 | 2.22E-12 |
| At_PAPS3 | SRR1946188_contig_1459 | 3.59976 |
| At_PAPS3 | SRR1946188_contig_8394 | 6.43E-25 |
| At_PAPS3 | SRR1946188_contig_8394 | 3.41E-13 |
| At_PAPS3 | SRR1946188_contig_8394 | 1.26E-05 |
| At_PAPS3 | SRR1946188_contig_8394 | 0.000552 |
| At_PAPS3 | SRR1946188_contig_4292 | 3.67E-20 |
| At_PAPS3 | SRR1946188_contig_4292 | 3.67E-20 |
| At_PAPS3 | SRR1946188_contig_4292 | 3.67E-20 |
| At_PAPS3 | SRR1946188_contig_4292 | 7.75E-10 |
| At_PAPS3 | SRR1946188_contig_4292 | 7.75E-10 |

C. Depiction of the results for reassembly of strain CS76769. Different sections are as described in Supplemental Fig. 4.

| Query sequence | Hit | E-value |
| --- | --- | --- |
| At_PAPS3 | SRR1946283_contig_2965 | 9.2E-143 |
| At_PAPS3 | SRR1946283_contig_2965 | 9.2E-143 |
| At_PAPS3 | SRR1946283_contig_2965 | 9.2E-143 |
| At_PAPS3 | SRR1946283_contig_2965 | 9.2E-143 |
| At_PAPS3 | SRR1946283_contig_2965 | 9.2E-143 |
| At_PAPS3 | SRR1946283_contig_2965 | 9.2E-143 |
| At_PAPS3 | SRR1946283_contig_2965 | 2.7E-106 |
| At_PAPS3 | SRR1946283_contig_2965 | 2.7E-106 |
| At_PAPS3 | SRR1946283_contig_5291 | 2.81E-27 |
| At_PAPS3 | SRR1946283_contig_5291 | 1.34E-10 |
| At_PAPS3 | SRR1946283_contig_5291 | 2.13235 |
| At_PAPS3 | SRR1946283_contig_4833 | 9.51E-27 |
| At_PAPS3 | SRR1946283_contig_4833 | 9.51E-27 |
| At_PAPS3 | SRR1946283_contig_4833 | 4.21E-13 |
| At_PAPS3 | SRR1946283_contig_4833 | 3.70901 |
| At_PAPS3 | SRR1946283_contig_2258 | 3.71E-20 |
| At_PAPS3 | SRR1946283_contig_2258 | 3.71E-20 |
| At_PAPS3 | SRR1946283_contig_2258 | 3.71E-20 |
| At_PAPS3 | SRR1946283_contig_2258 | 7.86E-10 |
| At_PAPS3 | SRR1946283_contig_2258 | 7.86E-10 |
| At_PAPS3 | SRR1946283_contig_3004 | 1.42E-13 |
| At_PAPS3 | SRR1946283_contig_3004 | 0.000586 |
| At_PAPS3 | SRR1946283_contig_6298 | 3.33E-13 |
| At_PAPS3 | SRR1946283_contig_6298 | 1.26E-05 |
| At_PAPS3 | SRR1946283_contig_6298 | 7.33E-05 |
